# Network Comparison of Inflammation in Colorectal Cancer and Alzheimer's Disease

**DOI:** 10.1155/2015/205247

**Published:** 2015-07-26

**Authors:** Sungjin Park, Seok Jong Yu, Yongseong Cho, Curt Balch, Jinhyuk Lee, Yon Hui Kim, Seungyoon Nam

**Affiliations:** ^1^New Experimental Therapeutics Branch, National Cancer Center, Goyang-si, Gyeonggi-do 410-769, Republic of Korea; ^2^Supercomputing R&D Center, Korea Institute of Science and Technology Information (KISTI), Daejeon 305-806, Republic of Korea; ^3^Bioscience Advising, Indianapolis, IN 46227, USA; ^4^Korean Bioinformation Center (KOBIC), Korea Research Institute of Bioscience and Biotechnology, Daejeon 305-806, Republic of Korea

## Abstract

Recently, a large clinical study revealed an inverse correlation of individual risk of cancer versus Alzheimer's disease (AD). However, no explanation exists for this anticorrelation at the molecular level; however, inflammation is crucial to the pathogenesis of both diseases, necessitating a need to understand differing signaling usage during inflammatory responses distinct to both diseases. Using a subpathway analysis approach, we identified numerous well-known and previously unknown pathways enriched in datasets from both diseases. Here, we present the quantitative importance of the inflammatory response in the two disease pathologies and summarize signal transduction pathways common to both diseases that are affected by inflammation.

## 1. Introduction

Epidemiological evidence has revealed an inverse incidence between Alzheimer's disease (AD) and cancer that increases exponentially among aged cohorts [1, 2]. However, despite the clear differences in the etiology of the two diseases, including the premature death of neurons in AD and evasion of apoptosis in cancer, it has been suggested that common signaling pathways are involved in the two age-associated diseases [3]. Molecular comparative surveys of the two disease states have led to speculation of roles for p53 and the Wnt signaling pathway in both cancer and AD [4]. However, a global transcriptomic network comparison between the two diseases has yet to be completed [2].

Of interest, immune response is intimately related to both diseases [5–7]. In fact, based on an early colorectal cancer (CRC) transcriptome dataset [8], our previous study [9] found immunosuppression and immune cell infiltration even within normal-appearing cells in CRC patients. Similarly, in the brain, microglia and astrocytes involved in inflammation play a critical role in neurodegeneration [6, 7].

Despite continuous efforts to understand the individual molecular mechanisms of the two diseases, distinction of the global effects of immune response toward specific signal transduction usage in the two diseases has not been established. Here, we systematically inspected the two diseases representing phenotypically opposite cell fates, death and survival, by utilizing functional enrichment analysis and a systems biology approach [9]. This functional enrichment indicated that inflammatory response was significantly involved in both diseases. Subsequently, we found, by the systems biology approach, that various pathways within each disease network were comprised of common inflammation-associated genes.

## 2. Materials and Methods

### 2.1. Functional Enrichment Comparison of CRC and AD

**Figure 1:**
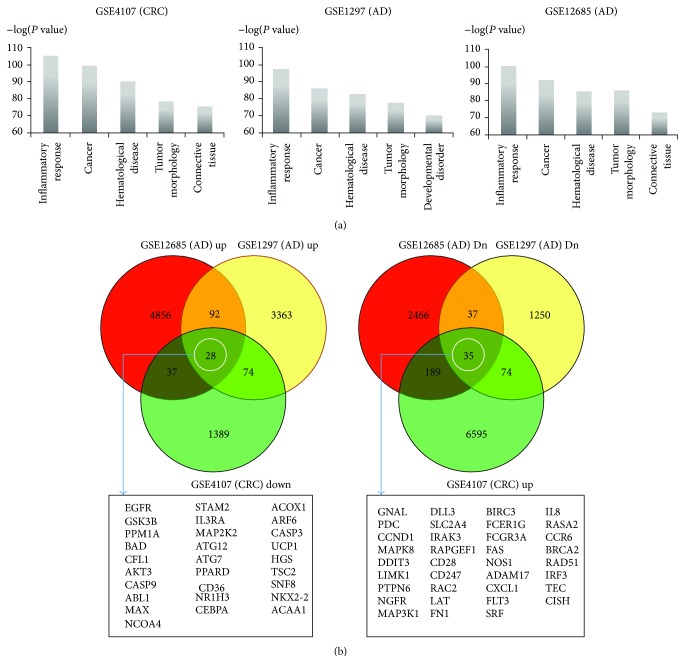
IPA functional enrichment of the CRC and the AD datasets. (a) Top 5 functional categories from “Diseases and Functions” ontology for the datasets are represented. The *y*-axis represents the minus logarithms of the *P* values. The higher the value on the *y*-axis is, the more statistically significant it becomes. The *x*-axis represents the functional categories. (b) The common genes inversely expressed between the two diseases are indicated by white ovals (see details in Section 2). In the Venn diagrams, “GSE12685 (AD) Dn” is the downregulated gene set in AD patients versus controls. “GSE12685 (AD) Up” is the upregulated gene set in AD patients versus controls. The notation is similar to the GSE1297 (AD) dataset and the GSE4107 (CRC) dataset.

Throughout the paper, we compared one colorectal cancer (CRC) dataset (GEO accession GSE4107) [8] with two AD datasets (GEO accessions GSE1297, GSE12685) [10, 11] from GEO (see details in Supplementary Table S1 in Supplementary Material available online at http://dx.doi.org/10.1155/2015/205247). We used Ingenuity Pathway Analysis (IPA, Qiagen, Valencia, CA, USA) to inspect functionally enriched terms within the IPA “Diseases and Functions” ontology, revealing the top 5 significant terms for the three datasets (Figure 1(a)). For functional enrichment analysis, we uploaded the expression fold-changes of all the genes for the three datasets into IPA: in the CRC dataset, the expression fold-changes of patients versus controls were obtained and in AD, the fold-changes of AD patients versus controls were obtained.

Since cancer and AD are phenotypically opposite (cell survival versus cell death), we obtained oppositely expressed common genes between the two diseases. Based on all the genes' fold-changes from the three datasets, we obtained the common genes as shown in Figure 1(b).

### 2.2. Network Construction of CRC and AD

For generating networks from the three datasets, we applied our previous subpathway-based systems biology approach [9]. In brief, KEGG pathways were decomposed to all their possible paths (i.e., subpathways). In a given dataset, we applied a statistical test to each subpathway to determine whether the gene expression levels agreed with edge types (e.g., activation, inhibition) of the subpathway. Subsequently, in the dataset, we gathered the statistically significant subpathways (*P* values <0.05) that comprised the network.

## 3. Results and Discussion

### 3.1. Overview

While cancer and AD are two of the most common diseases worldwide (15.6 million versus 7.7 million new cases per year) relating to aging, their phenotypes are opposite: cell death (neurons) in AD versus survival (mostly epithelial cells) in cancer. Also, AD patients are less susceptible to cancer and vice versa [1]. Consequently, we aimed at understanding changes at the molecular level between the two diseases. First, we inspected functional enrichment comparison of a cancer dataset (from our previous study) and the two AD datasets. Second, due to the involvement of inflammation in both pathologies [12, 13], we aimed to identify global network differences between the two diseases to possibly identify differential inflammation environments and differential chemokine/cytokine receptor usages. For this purpose, we selected colorectal cancer (CRC) as the cancer dataset to extend our previous result [9]. We also obtained the two independent AD datasets from GEO (Supplementary Table S1).

### 3.2. Functional Enrichment Comparison of CRC and AD: Inflammation-Related Genes

We used Ingenuity Pathway Analysis (IPA) to perform functional pathway enrichment of early CRC and AD. IPA reported the top 5 functional categories from its “Diseases and Functions” ontology. In Figure 1(a), inflammatory response-related genes, as well as cancer-associated genes, were significantly enriched in the CRC and the AD datasets.

Figure 1(b) shows common genes that were inversely expressed between the two phenotypically opposite diseases. Considering that the biopsy tissues for the datasets contain immune cells, inflammatory response is reasonable for functional enrichment.

Out of the common genes in Figure 1(b),* ARF6* was upregulated in the AD datasets but downregulated in the CRC dataset. ARF6, a small GTPase [14–16], regulates early endosome internalization of the protease BACE1, Beta-Site APP-Cleaving Enzyme 1. This internalization enables BACE1 to encounter and cleave intracellular amyloid precursor proteins (APPs), leading to amyloidogenic processing for the accumulation A*β* dimers in neurons, a hallmark of AD pathology [17].

*CCR6* (in Figure 1(b)) was upregulated in CRC but downregulated in both AD datasets. CCR6 is an important surface marker of immunosuppressive immune cells in the CRC tumor microenvironment [18]. Regulatory T cells (T_Reg_ cells) expressing CCR6 are recruited to a tumor mass by tumor-associated macrophages (TAMs), and tumor development is enhanced by CCR6 binding to its ligand CCL20 (CRC 1.721-fold of overcontrol in the GSE4107 dataset) secreted by tumor cells [18]. This scenario agrees with our previous result, indicating T_Reg_ cell infiltration into normal-appearing mucosa in CRC patients [9]. Considering that T and B cells do not exist in brain, the low expression of the T_Reg_ cell surface markers in AD patients is not surprising.

We further dissected the common genes (28 and 35 genes in white circles in the Venn diagram in Figure 1(b)) in terms of inflammation, considering that inflammatory response was the highest enrichment in all three datasets. For this purpose, we selected several terms involved in inflammation from the IPA “Diseases and Functions” ontology (see the terms and entries in Supplementary Table S2). Out of the genes common to the three datasets, 16 were oppositely expressed between the two diseases in terms of IPA inflammation-related terms (Table 1).

### 3.3. Network Construction of CRC and AD

Next, we constructed molecular networks for the two diseases. By applying our previous systems biology method to the three disease datasets, we obtained CRC and AD pathogenesis networks (Supplementary Figures S1–S3). We summarized the most significant 100 subpathways for each network (Supplementary Tables S3–S5) in order to see the signaling in detail. These subpathways were assigned to various pathways in CRC and AD (Supplementary Tables S3–S5), suggesting that, in addition to inflammatory response inferred by our functional enrichment comparison, those pathways (not assigned to inflammation) remain largely unexplored in CRC or AD. Of interest, we found pathways previously unassociated with the two diseases, including Hedgehog signaling, axon guidance, ECM-receptor interaction, and WNT signaling (Table 3). In CRC, WNT3 facilitates crosstalk between the Hedgehog and Wnt signaling pathways (Table 3). Similarly, ECM-receptor interaction was oppositely regulated between the two diseases.

### 3.4. Opposite Signaling Pathway Expression between CRC and AD by Inflammation-Related Genes

The AD datasets were prepared from frontal cortex synaptoneurosomes and hippocampi. Both brain regions include neurons, as well as astrocytes and microglia [19, 20]. In our previous analysis [9] of the CRC dataset, immune cells were infiltrating. Considering immune cell involvement in the two diseases and their two opposite phenotypes, different inflammation-related molecule usage in signaling is self-evident.

So, we inspected the 16 genes' (in Table 1) differential usage of the CRC and AD networks (from Supplementary Figures S1–S3).  Table 2 indicates that 16 genes were involved in extensive signaling transduction in both the CRC and AD networks, and all were inversely expressed between the two diseases.

Out of the 16 gene products, CD36 (a class B scavenger receptor) was found in microglia and vascular endothelial cells of AD patient brains [21]. Activation of CD36 and PPAR delta (gene symbol: PPARD, upregulated in both AD datasets in Table 2) resulted in FoxO1 activation in a functional study of muscle cells [22]. Considering that microglia are activated by FoxO1 [23], the two genes (*CD36* and* PPARD*) could be involved in inflammation of AD patient brains.

Another intriguing observation was the opposite expression of a cell growth (antiapoptosis) gene,* MAPK*, which was upregulated in CRC and downregulated in AD, while two apoptosis pathways genes,* FAS* (part of the extrinsic apoptosis pathway) and* BAD*, showed the opposite pattern (up in AD and down in CRC) (Table 2). This apoptosis versus cell survival relationship has been previously postulated to explain the inverse risk correlation between malignant and neurodegenerative diseases.

## 4. Conclusions

In general, single gene expression analysis looks into highly differentially expressed genes under a certain cutoff (e.g., *P* value, fold-change). However, in real biological problems, signaling proteins involved in phenotype differences may not show a drastic expression-level change [9, 24]. Also, considering that phenotype change or disease results from dysregulation of complex relationships between biological components [25, 26], a strict cutoff usage in single gene analysis can miss signal flow. For example, some biological entities belonging to the flow would be filtered out under a certain cutoff. Along that line, we applied our previous systems biology method [9] for describing the interdependency underlined in the diseases. In summary, we found that inflammatory response was a very important mechanism in two diseases of opposite phenotypes, that is, cancer (cell survival) and Alzheimer's disease (cell death). The inflammation-related common genes between the diseases regulated opposite gene expression in various cell signaling in the two-disease networks. In other words, the inflammation-related genes in Table 2 utilized different pathways according to the disease states, leading to different signaling transductions. Further investigation of such networks could provide knowledge into the immunological bases for the progression of both of these devastating diseases.

## Supplementary Material

Supplementary Material contains five supplementary tables (Tables S1 through S5) and three supplementary figures (Figures S1 through S3). Also it includes their legends.

## Figures and Tables

**Table 1:** Inflammation-associated genes common to both AD and CRC show opposite expression patterns. The 16 oppositely expressed common genes (in Figure 1(b)) between AD and CRC were assigned to inflammation-associated functional terms in IPA.

Functional category	Downregulated in AD and upregulated in CRC	Upregulated in AD and downregulated in CRC
Chemokine	PTPN6^+∗#^, IRAK3^+∗#^, FLT3^+∗#^	BAD^+∗#^, CD36^+∗#^

Inflammation relating to CRC	DDIT3^+∗#^, FAS^+∗#^, IRF3^+∗#^	

Inflammation relating to brain	CCR6^+∗#^, CD28^+∗#^, DDIT3^+∗#^, FAS^+∗#^, FCER1G^+∗#^, NGFR^+∗#^	PPARD^+∗#^

Cytokines relating to cancer	CD28^+∗#^, FN1^+∗#^	ABL1^+∗#^, EGFR^+∗#^

Cytokines relating to brain		CD36^+∗#^

^+^Genes detected in the CRC network from GSE4107 dataset.

^*^Genes detected in the AD network from GSE1297 dataset.

^#^Genes detected in the AD network from GSE12685 dataset.

**Table 2:** KEGG pathways associated with the 16 oppositely expressed common genes (in Table 1) in the AD and the CRC networks. From the AD and the CRC networks, pathway information of the 16 genes was obtained. The 16 genes were inversely expressed in the pathways between the AD and the CRC networks.

Gene symbols	Pathways	CRC (GSE4107)	AD (GSE12685)	AD (GSE1297)
PTPN6	hsa04662_B_cell_receptor_signaling_pathway; hsa04630_Jak-STAT_signaling_pathway; hsa05140_Leishmaniasis	Up	Down	Down
IRAK3	hsa04722_Neurotrophin_signaling_pathway			
FLT3	hsa05221_Acute_myeloid_leukemia			
DDIT3	hsa04010_MAPK_signaling_pathway			
FAS	hsa04115_p53_signaling_pathway; hsa04650_Natural_killer_cell_mediated_cytotoxicity			
IRF3	hsa04622_RIG-I-like_receptor_signaling_pathway; hsa04623_Cytosolic_DNA-sensing_pathway			
CCR6	hsa04060_Cytokine-cytokine_receptor_interaction; hsa04062_Chemokine_signaling_pathway			
CD28	hsa04660_T_cell_receptor_signaling_pathway; hsa05416_Viral_myocarditis			
FCER1G	hsa04650_Natural_killer_cell_mediated_cytotoxicity			
NGFR	hsa04722_Neurotrophin_signaling_pathway			
FN1	hsa04512_ECM-receptor_interaction			

BAD	hsa04510_Focal_adhesion; hsa05223_Non-small_cell_lung_cancer; hsa05210_Colorectal_cancer	Down	Up	Up
CD36	hsa03320_PPAR_signaling_pathway; hsa04512_ECM-receptor_interaction			
PPARD	hsa05221_Acute_myeloid_leukemia; hsa04310_Wnt_signaling_pathway			
ABL1	hsa04012_ErbB_signaling_pathway; hsa04722_Neurotrophin_signaling_pathway			
EGFR	hsa05214_Glioma; hsa04012_ErbB_signaling_pathway			

**Table 3:** Subpathways previously not associated with the two diseases. These subpathways were selected from the most significant 100 subpathways in each network. Subpathway (linear signaling flow) with fold-change (the numeral in parenthesis) of the disease group over the control group is represented in each dataset. The most significant 100 subpathways for each dataset are provided in Supplementary Tables S3–S5. The notation in the flow is “B <- A: A activates B” and “B ∣- A: A represses B.”

KEGG pathway	GSE4107 (CRC) subpathway; *P* value	GSE1297 (AD) subpathway; *P* value	GSE12685 (AD) subpathway; *P* value
Hedgehog signaling (hsa04340)	PTCH1 (1.863) <- GLI2 (2.878) ∣- CSNK1G1 (0.587); 0.000035		PTCH2 (0.938) <- GLI3 (0.682) ∣- GSK3B (1.513); 0.0015
	WNT3 (3.147) <- GLI2 (2.878) ∣- CSNK1G1 (0.587); 0.000223		

Axon guidance (hsa04360)		PAK3 (0.732) <- RAC1 (0.943) ∣- PLXNB3 (1.627) <- SEMA4C (1.283); 0.0008	CFL1 (1.157) ∣- LIMK1 (0.896) <- PAK4 (0.871) <- RAC3 (0.892) <- PLXNA3 (0.954) <- FES (0.841); 0.0011

WNT signaling (hsa04310)	JUN (4.179) <- TCF7L1 (2.735) <- CTNNB1 (2.562) ∣- GSK3B (0.735) ∣- DVL3 (1.608) <- FZD10 (6.256) <- WNT3 (3.147) <- PORCN (1.279); 0.000114		
	JUN (4.179) <- TCF7L1 (2.735) <- CTNNB1 (2.562) ∣- GSK3B (0.735) ∣- DVL3 (1.608) <- APC2 (2.201) <- AXIN2 (2.307) <- CSNK1A1 (1.963); 0.00016		

Pathways in cancer (hsa05200)	MMP2 (3.031) <- JUN (4.179) <- MAPK1 (2.425) <- MAP2K1 (1.162) <- ARAF (4.631) <- HRAS (1.027) <- SOS1 (1.624) <- GRB2 (1.613) <- IGF1R (2.299) <- IGF1 (2.529); 0.000022		

ECM-receptor interaction (hsa04512)	SDC2 (3.091) <- TNC (9.557); 0.000026	SDC3 (0.849) <- COL5A2 (0.162); 0.003	SDC1 (0.865) <- COL3A1 (0.865); 0.0017
	SDC2 (3.091) <- FN1 (5.594); 0.000125		

Neurotrophin signaling (hsa04722)			BAD (1.279) ∣- AKT2 (0.856) <- PDK1 (0.943) <- PIK3CD (0.576) <- GAB1 (0.997) <- SHC2 (0.844) <- NTRK1 (0.945) <- NTF3 (0.784); 0.0008
