# Supplementary material for: Network Comparison of Inflammation in Colorectal Cancer and Alzheimer's Disease
Source: Biomed Res Int. 2015 Jul 26;2015:205247. doi: 10.1155/2015/205247 (PMC4529906; doi:10.1155/2015/205247)
Supplement: Supplementary file 1 — Supplementary Material contains five supplementary tables (Tables S1 through S5) and three supplementary figures (Figures S1 through S3). Also it includes their legends. [file 205247.f1.pdf]

Network comparison of inflammation in colorectal cancer and Alzheimer's disease

Park *et al*.

Supplementary Information

Contents

Tables S1-S5

Supplementary Figure legends

Figures S1-S3

References

Table S1. Data description used in this study.

GEO accession number	Data description	Reference
GSE4107 (CRC)	Young CRC patients (12 samples) with non-FAP or non-hereditary nonpolyposis colorectal cancer, and healthy controls (age <=50 years old) (10 samples) were compared.	[1]
GSE12685 (AD)	Synaptoneurosomes (in frontal cortex) of incipient AD (IAD) patients (6 samples) and those of controls (8 samples) were compared.	[2]
GSE1297 (AD)	Hippocampal gene expression of nine controls and 22 AD subjects were compared.	[3]

Table S2. The IPA terms and their entries relating to inflammation, cytokines and chemokines.

Subjects	IPA terms	Entries (including chemicals)
Inflammation relating to brain	Inflammation of nervous system<br>Inflammation of central nervous system<br>Inflammation of cerebral cortex<br>Inflammation of nervous tissue<br>Inflammation of neuroglia<br>Inflammation of neurons<br>Inflammation of microglia<br>Inflammation of astrocytes<br>Inflammation of cerebellar granule cell<br>Inflammation of neuroendocrine cells	2-methoxyestradiol; 3-aminobenzamide; 3-hydroxybutyric acid; 3-hydroxykynurenine; 6-hydroxydopamine; 7-valent pneumococcal vaccine; 15-deoxy-delta-12; 14 -PGJ 2; 17-alpha-ethinylestradiol; ABCG2; ACKR2; ACKR4; acyclovir; adenosine; ADIPOQ; ADORA2A; ADORA2B; AGER; AHR; ALCAM; ALOX5; ALS2; Ampa Receptor; ancrod; APP; ARID5A; ARRB1; atorvastatin; aurintricarboxylic acid; B2M; BATF; BCL2L1; beta-estradiol; BHLHE40; brivudine; BTLA; BTN1A1; butyric acid; C3; C5; C6; C10orf54; C3AR1; C4A/C4B; calcitriol; CALR; CASP1; CASP4; CBLB; Ccl2; CCL3; CCL3L3; CCR1; CCR2; CCR4; CCR6; CCR8; Cd1; CD3; CD4; CD5; CD19; CD24; CD28; CD40; CD44; CD47; CD74; CD80; CD86; CD200; CD274; CD276; CD1D; CD300LF; CD40LG; Cd59a; CDK5R1; celecoxib; ceramide; cerebroside 3-sulfate; chickenpox vaccine; cholecalciferol; choline; CIITA; CLEC4D; CLEC4E; CLEC5A; CNR2; CNTF; copper; CORO1A; CORT; corticosteroid; corticosterone; CRH; CRP; CSF2; CSF3; CSMD1; CST3; CTLA4; CTSB; cuprizone; CX3CR1; CXCL10; cyanocobalamin; DDIT3; dehydroisoandrosterone; delta-9-tetrahydrocannabinol; deoxycorticosterone acetate; dexamethasone; DLL1; DLL4; DPP4; DryVax smallpox vaccine; DUSP1; DUSP10; E. coli B5 lipopolysaccharide; EBI3; EGR2; EIF2AK3; ELAVL1; ELF1; ENTPD7; enzastaurin; EPHA4; EPHB6; epigallocatechin-gallate; ESR1; ESR2; estriol; estrogen; etoposide; F2RL1; FAS; FASLG; fasudil; FCER1G; FCGR1A; FCGR1B; FCGR2A; FCGR2B; FCGR2C; FCGR3A/FCGR3B; fenofibrate; fingolimod; FKBP1A; FUS; GADD45B; galactosylceramide-alpha; ganciclovir; gemfibrozil; GFAP; GFI1; GHRH; GHRHR; glutathione ethyl ester; GRIA3; HAVCR2; Havrix; HCST; HDC; HEXB; HIF1A; HLA-A; HLA-DMA; HLA-DQA1; HLA-DQB1; HLA-E; HPX; HRH1; HRH2; HRH3; HRH4; HSPA5; hyaluronidase; ICOS; ID2; IDO1; IFI30; IFN Beta; IFNA1/IFNA13; IFNAR1; IFNB1; IFNE; IFNG; IFNGR1; IGF1; IgG; Igh (family); IGHG1; Ighg2b; IGHM; IKBKB; IKBKG; IL4; IL6; IL9; IL10; IL13; IL18; IL21; IL23; IL25; IL27; IL-1R; IL12 (complex); IL12A; IL12B; IL12RB2; IL17A; IL17C; IL18BP; IL18R1; IL1B; IL1R1; IL1RN; IL21R; IL23A; IL23R; IL27RA; IL2RA; IL6ST; IL9R; imatinib; Immunoglobulin; Interferon alpha; IRAK1; IRAK4; IRF1; IRF4; IRF9; Irgm1; ITGA9; ITGAL; ITGAX; ITGB8; JAG1; kainic acid; KCNA3; KCNK2; KIT; KLF4; KLRB1; L-826; 141; L-tryptophan; LCP2; LEP; lestaurtinib; let-7; LGALS1; LGALS3; LGI1; LIF; linsidomine; lipopolysaccharide; lovastatin; LTBR; luteolin; LYN; MAML1; MAP4K1; MAPK3; MAPK11; MAPK14; MAVS; MBP; MDK; methotrexate; methylprednisolone; MGAT5; MGAT5B; MGLL; MHC II; MIF; mifepristone; MIR124; mir-155; mir-326; miR-124-3p (and other miRNAs w/seed AAGGCAC); mitoxantrone; MME; MMP2; MMP8; MMP9; MOG; montelukast; MPO; MR1; Mt1; Mt2; MTOR; MYD88; MYLK; natalizumab; NCF1; neuropeptide Y; NFATC2; NFKB2; NFkB (complex); NGF; NGFR; nitric oxide; NLRP3; NLRP10; NOS2; NOTCH1; NOTCH2; NP 031112; NPY; NR3C1; NR4A2; NRG1; NS-398; NT5E; P38 MAPK; PDCD1; PDCD4; PDCD1LG2; PECAM1; PELI1; peptidoglycan; peramivir; pioglitazone; PLA2G4A; PLAT; PLP1; PPARA; PPARD; PPARG; PPID; PPP3CA;

		PPP3CB; PPP3CC; PPT1; PRDM1; prednisolone; PRF1; PRKAA; PRKCA; PRKCQ; PROC; prostaglandin E2; PS-1145; PSEN1; PTAFR; PTGER2; PTGER4; PTGS2; PTPN22; PTPRC; PTPRZ1; purine analogs; PYCARD; quinolinic acid; rabies vaccines; Rag; RBPJ; REL; RELA; RFTN1; rituximab; RNF128; rolipram; roquinimex; RORA; RORC; RT1-B; S1PR1; scyllo-inositol; SDC1; Secretase gamma; SELL; SEMA4A; SEMA4D; SGPL1; sirolimus; SIRT1; SIT1; SMAD2; SOD1; SPI1; spironolactone; SPN; SPP1; SREBF2; SST; STAT1; STAT4; STAT6; STK17B; tacrolimus; tamibarotene; taurine; TBX21; Tcf7; TCR; Tcrd; TGAL copolymer; TGFB1; TH1 Cytokine; TH2 Cytokine; thioctic acid; TICAM1; TIGIT; Timd2; TIMP1; TLR3; TLR4; TLR9; Tlr; TNF; Tnf receptor; TNFRSF4; TNFRSF14; TNFRSF21; TNFRSF10B; TNFRSF12A; TNFRSF1A; TNFRSF1B; TNFRSF6B; TNFSF4; Tnfsf9; TNFSF10; TNFSF14; TNFSF15; TOB1; TP53BP2; TRAF3; TRAF3IP2; tretinoin; triamcinolone; trichostatin A; TRPM4; TSPAN2; TWIST1; TYMP; TYROBP; UBASH3A; UNC93B1; UPP1; uric acid; ursolic acid; USP18; valacyclovir; valproic acid; VCAM1; VDR; Vegf; VEGFC; VIM; vitamin D; vorinostat; VTCN1; VWF; Wld; xaliproden; XBP1; XIAP; yellow fever vaccine; zafirlukast; zymosan
Inflammation relating to CRC	Inflammation of tumor<br>Inflammation of tumor cell lines<br>Inflammation of colonic mucosa<br>Chronic inflammation of colon<br>Colitis<br>Eosinophilic inflammation of colon<br>Inflammation-associated cancer<br>Inflammation of tumor<br>Inflammation of benign tumor<br>Inflammation of adenoma	2,4-dinitrobenzenesulfonic acid; 15-keto-13,14-dihydroprostaglandin E2; [D-Ala2,N-Me-Phe4,Gly5-ol]-Enkephalin; abatacept; ABCB1; ABCC4; acetic acid; adalimumab; ADAMTS12; ADORA2A; AGR2; AHR; AKT1; AKT2; Akt; ALB; ALOX5; ALOX12; anakinra; anthracycline; anthracycline/taxoid derivative; ANXA1; APOA4; ATG16L1; ATM; atorvastatin; ATP11C; AXL; azathioprine; azoxymethane; B2M; B3GNT6; balsalazide; BATF; betamethasone; betamethasone acetate/betamethasone phosphate; betamethasone phosphate; BSN; budesonide; BW 245C; C3; CALCA; CALCB; capecitabine; carboplatin; CASP1; CCL2; Ccl2; CCL11; CCL18; CCR2; CCR5; CD3; CD40; CD44; CD47; CD69; CD80; CD86; CD274; CD40LG; CEACAM1; CEBPE; celecoxib; CFB; CFH; chloroquine; ciprofloxacin; CKB; CKM; CKMT1A/CKMT1B; COL1A1; COL1A2; CORT; corticosteroid; corticotropin; cortisone acetate; CR2; CR1L; creatine; CRLF2; CRP; CSF2; CSF1R; CTLA4; curcumin; CXCL2; CXCL3; CXCL5; CXCL8; CXCL10; cyclophosphamide; cyclosporin A; CYLD; CYP11A1; CYP11B1; DDIT3; DDX58; deligoparin; dexamethasone; dexamethasone 21-acetate; dexamethasone phosphate; dexanabinol; dexrazoxane; dextran sulfate; DMBT1; docosahexaenoic acid; doxorubicin; doxycycline; EBI3; EDN1; EMR1; enoxaparin; ENTPD1; epaxal beRNA; epirubicin; ERBB2; EREG; ERK; ERN2; ESR2; etrolizumab; F10; F2R; F2RL1; FAAH; FAS; FCER1A; FERMT1; ferric carboxymaltose; ferrous sulfate; FGFR2; FKBP1A; folic acid; formaldehyde; FPR2; FUT7; gabapentin; GCNT3; gemcitabine; glucosylceramide; Gm21596/Hmgb1; GNAI2; golimumab; GPX1; GPX2; grape seed extract; GTPBP1; HGF; histamine; HLA-B27; holmium-166 acetylacetonate; HPGD; HPGDS; HSD11B1; HSF1; HSF2; Hsp70; HSP90B1; HTR7; HU-210; hydrocortisone; hydrocortisone cypionate; hydrocortisone phosphate; hydrocortisone succinate; hydrocortisone/prednisone; I kappa b kinase; ICAM1; IFNB1; IFNG; IgG1; IGHG4; IGHM; IL2; IL4; IL6; IL7; IL10; IL13; IL18; IL22; IL23; IL24; IL25; IL37; IL-1R; IL10RA; IL10RB; IL11RA; IL12 (complex); IL12 (family); IL12A; IL12B; IL13RA2; IL17A; IL17C; IL1B; IL1R1; IL1RN; IL22RA2; IL23A; IL23R; IL27RA; IL2RA; IL6 receptor; IL6R; IL6ST; Immunoglobulin; indisulam; indomethacin; infliximab; Integrin; ipilimumab; IRF3; iron sucrose; ITGA1; ITGB3; ITGB7; ITGB8; JAK1; JAK2; JAK3; JAK; KRAS; KSR1; lactosylceramide;

		LBP; LCN2; LGALS4; lipopolysaccharide; low molecular weight heparin; LPAM; LRRK2; LTB; LTBR; LY96; LY294002; Map3k7; MC2R; MEP1A; MERTK; mesalamine; methotrexate; methylprednisolone acetate; methylprednisolone succinate; metronidazole; MME; MMP7; MMP9; monoclonal antibody; MPO; MS4A1; MST1; MTOR; MUC1; MYD88; N-(3-(aminomethyl)benzyl)acetamidine; N-nitro-L-arginine methyl ester; naloxone methiodide; naltrexone; NCF1; NCF4; NFE2L2; NFIL3; NFKB1; NFKBIA; NFKBID; nitazoxanide; NKX2-3; NLRP3; NLRP6; NLRP12; NOD1; NOD2; NOS2; NOS3; NR1H4; NR3C1; NR5A2; NRG4; olsalazine; OPRD1; OPRK1; OPRM1; Oxi-4503; paclitaxel; Pam3-Cys-Ser-Lys4; PD98059; PDCD1; PDE4A; PDE4B; PDE4C; PDE4D; peptidoglycan; pertuzumab; PGLYRP2; PGLYRP3; PGLYRP4; PHB; phenylbutazone; phosphatidylinositol; PI3K (family); PI3KÎ³; PIK3CD; PIK3CG; PIK3R1; pimagedine; PIP; piroxicam; plerixafor; PLG; PON1; POR; PPARG; PPP3CA; PPP3CB; PPP3CC; pralnacasan; pravastatin; prednisolone; prednisolone phosphate; prednisone; prinaberel; PROC; PROCR; propionyl-L-carnitine; PROS1; PTGER4; PTGS1; PTGS2; PTPN11; PTPN22; PYCARD; RAG1; RAG2; ranitidine; RASGRP4; RELA; Retnla; RETNLB; rifampin; rimonabant; rituximab; RORC; RPTOR; RUNX3; RXRA; S100A8; S1PR1; S1PR4; SELE; SELP; SERPINE1; SIGIRR; SIGMAR1; silibinin; SIRT6; SLPI; SMAD3; SMAD7; SOCS1; SOCS3; SOD1; SPP1; STAT1; STAT3; STAT4; STAT6; STAT5A; STAT5B; STX2; sucralfate; sulfasalazine; tacrolimus; tamoxifen; taxoid derivative; TBX21; Tcrd; tetomilast; TFF1; TFF3; TGFB1; TGFBR2; thalidomide; TKTL1; TLR1; TLR2; TLR3; TLR4; TLR5; TLR9; Tlr; TNF; TNFAIP3; TNFRSF14; TNFRSF11B; TNFRSF1A; TNFSF12; tofacitinib; TP53; TP53INP1; TPMT; TRA; TRAF3IP2; trastuzumab; TRB; TREM1; TREX1; TRG; triamcinolone; triamcinolone acetonide; trinitrobenzenesulfonic acid; triptolide; troglitazone; TRPM8; TRPV1; TYK2; ustekinumab; valproic acid; VDR; vedolizumab; VEGFA; visilizumab; vorinostat; WAS; WNT4; XBP1; ZNF148
Cytokines relating to brain	Chemotaxis of GABAergic neurons<br>Chemotaxis of astrocytes<br>Chemotaxis of axons<br>Chemotaxis of brain cells<br>Chemotaxis of central nervous system cell<br>Chemotaxis of cerebellar granule cell<br>Chemotaxis of cerebral cortex cells<br>Chemotaxis of cortical astrocytes<br>Chemotaxis of granule cells<br>Chemotaxis of hippocampal cells<br>Chemotaxis of hippocampal neurons<br>Chemotaxis of microglia<br>Chemotaxis of nervous tissue cell lines<br>Chemotaxis of neurites	Actin; ADP; ANGPT2; APP; ARTN; BDNF; Bmp7-Gdf7; bucladesine; C5; Ca2+; CAV1; CCL2; CCL19; CCL20; CCL21; CD36; CD47; CpG oligonucleotide; CSF1R; CX3CL1; CX3CR1; CXCL9; CXCL10; CXCL12; CXCR4; CYBB; cyclic AMP; cyclic GMP; EFNB1; EPHB2; ERBB2; FASLG; FGF2; FPR2; FYN; GAS6; GDNF; GFRA1; IL6; lipopolysaccharide; LRRK2; N-formyl-Met-Leu-Phe; NGF; NRCAM; NRP1; NRP2; NTF3; NTN1; palmitoyl-Cys((RS)-2,3-di(palmitoyloxy)-propyl)-Ala-Gly-OH; Pdgf (complex); peptidoglycan; phorbol myristate acetate; PI3K (complex); PICK1; Pkc(s); plasminogen activator; RGS3; RHOD; ROBO1; ROBO2; Sema3; SEMA3A; SEMA3B; SEMA3F; SIRPA; SLIT2; SMAD3; sphingosine-1-phosphate; TGFB1; TLR2; TNFRSF6B; UNC5A

	Chemotaxis of neuroblasts<br>Chemotaxis of neuroglia<br>Chemotaxis of neurons<br>Chemotaxis of sympathetic neurites<br>Chemtoaxis of granule cell precursors	
Cytokines relating to cancer	Chemotaxis of tumor cell lines<br>Chemotaxis of tumor cells<br>Chemotaxis of carcinoma cell lines<br>Chemotaxis of cancer cells<br>TH1 immune response [type 1 cytokine response]<br>Th2 immune response [type 2 cytokine response, Th2 cytokine response]	1-oleoyl-lysophosphatidic acid; 5,8,11,14-eicosatetraynoic acid; 6-n-octylaminouracil; ABL1; ACKR1; ACKR4; AGN194204; AKT1; alpha-GalCer-6''-(pyridin-4-yl)carbamate; aluminum hydroxide; ANXA2; APP; ARRB2; AXL; AZ12441970; baclofen; BCL3; BCL6; BCR; bleomycin; C3; C5; C5AR1; calpeptin; CALR; CAPN2; CBL; CCL1; CCL2; Ccl2; CCL3; CCL4; CCL5; CCL7; CCL8; CCL11; CCL13; CCL14; CCL15; CCL16; CCL17; CCL18; CCL19; CCL21; CCL22; CCL23; CCL24; CCL25; CCL26; CCL27; CCL3L1; CCR1; CCR2; CCR5; CCR8; CCR9; CD3; CD28; CD40; CD69; CD86; CD151; ceramide; chelerythrine; CLCN3; CLEC7A; Cofilin; COL4A3; COL4A3BP; Collagen Alpha1; Collagen type IV; CpG ODN 1668; CpG ODN 2216; CpG oligonucleotide; CREB3; CSF1; CSF2; CSK; CTSG; CX3CL1; CXCL3; CXCL8; CXCL9; CXCL10; CXCL12; CXCL13; CXCL14; CXCR1; CXCR3; CXCR4; cyclosporin A; cyclosporin H; damnacanthal; DEFB103A/DEFB103B; DOCK2; dopamine; DRD3; E. coli lipopolysaccharide; EBI3; EDN2; EGF; EGFR; ELF3; ERBB3; ETV4; FADD; FASLG; FBN1; FGF2; FIGF; FLT1; FLT3LG; FN1; FPR1; FPR2; GABA; galactosylceramide-alpha; GAS6; GDNF; geldanamycin; genistein; GNB1; GNB2; GPR132; GRB2; HBEGF; HGF; histamine; HLA-DQB1; HLA-DRB3; HMGB1; Hnp alpha; HOXA7; HRH1; HRH2; HRH4; ICAM1; ICOS; ICOSLG/LOC102723996; Ifn gamma; IFN type 1; IFN1@; IFNA1/IFNA13; IFNB1; IFNG; IFNL1; IGF1; IL3; IL4; IL5; IL6; IL7; IL9; IL10; IL13; IL15; IL18; IL25; IL33; IL12 (complex); IL12 (family); IL12A; IL12B; IL12RB1; IL12RB2; IL17A; IL18BP; IL1B; IL1R1; IL1RL1; IL23A; IL23R; IL27RA; IL4R; iloprost; indomethacin; Interferon alpha; IRAK1; IRF1; ITCH; ITGA1; ITGA2; ITGA6; ITGAV; ITGB1; ITGB3; ITK; JAG2; Jnk; JUN; K-252; KCNN4; KIT; KITLG; KNG1; lactose; Laminin; LCK; LDL; leukotriene D4; Lewis x antigen; LIF; LIFR; lipopolysaccharide; LPAR3; LTA; luminespib; LY294002; lysophosphatidic acid; lysophosphatidylcholine; mannosylated lipoarabinomannan; MAP2K3; MAP3K8; MERTK; mir-126; miR-126a-5p (and other miRNAs w/seed AUUAUUA); MOG; montelukast; MTOR; MTORC1; MYD88; N-acetylmuramyl-L-alanyl-D-isoglutamine; N-formyl-Met-Leu-Phe; N-nitro-L-arginine methyl ester; NCF1; NDFIP1; NEDD4; NFkB (complex); NLRP10; nocodazole; NOD1; NOD2; NOS2; NRG1; NRP1; NUP85; OSM; oxophenylarsine; P2RY2; P38 MAPK; p70 S6k; p85 (pik3r); Pam3-Cys; Pam3-Cys-Ser-Lys4; PARD3; PD98059; PDGF BB; peptidoglycan; PGAP3; PGF; phorbol esters; PI3K (complex); PI3K (family); piceatannol; PIK3CD; PIK3R1; pirinixic acid; Pka; PLA2G5; PLAU; PLAUR; PLD1; PLD2; plerixafor; PLG; poly rI:rC-RNA; POSTN; PPARG; PPIA; pranlukast; PRKCG; PRKCZ; PTEN; RADIL; RARRES2; RBP3; REL; RELB; resiquimod; RGS3; RHOU; ribavirin; RICTOR; RIPK2; RLN2; rosiglitazone; rosuvastatin; RXFP1; S1PR1; S1PR4; Sb202190; SB203580; SELPLG; SEMA3A; SHC1; SLC11A1; SLIT2; SMAD3; SMAD4; sodium alum; sodium orthovanadate; sphingosine-1-phosphate; SPHK1; SPTLC1; STAT3; STAT4; STAT6; stibogluconic acid; SYK; targinine; TBX21; Tcra-V14;

		TFF1; TGFB1; thioperamide; TICAM1; TLR2; TLR4; TLR9; TNF; TNFRSF8; TNFSF8; tosyllysine chloromethyl ketone; TRAF6; TRAF3IP2; trichosanthin; TRIP10; troglitazone; tyrphostin AG 1296; Ubiquitin; UTP; vanadate; VEGFA; vinpocetine; WASL; WDR26; wortmannin; Y 27632; Zfp35; zoledronic acid
Chemokines	Cytokine and chemokine mediated signaling pathway<br>Abnormal quantity of chemokine	ACKR1; ACKR4; AHR; BAD; BCL6; CAV1; CCL2; Ccl2; CCR2; Ccr2; CD36; CD74; CLCA1; CPB2; CTSC; CX3CL1; CX3CR1; CXCR2; DUOX1; DUOX2; EBI3; EDN2; ENPP2; EREG; FER; FKBP1A; FLT3; FOXP3; FYN; GHR; HCK; HRH4; Ierepo2; IFNAR1; IFNK; IFNLR1; Ifnz (includes others); Iigp1; Il3; IL5; IL6; IL10; IL13; IL10RA; IL10RB; IL11RA; IL12RB1; IL12RB2; IL13RA2; IL15RA; IL17RB; IL17RC; IL1A; IL1B; IL1R1; IL1R2; IL1RAP; IL1RAPL2; IL1RL2; IL1RN; IL21R; IL22RA1; IL22RA2; IL23R; IL2RB; IL2RG; IL31RA; IL36RN; IL5RA; IL6R; IL6ST; IRAK1; IRAK2; IRAK3; IRAK4; JAK1; JAK2; JAK3; KIT; KLF6; KRAS; LBP; LIFR; LILRB3; LYN; MEP1A; MMP9; MYD88; NLRP12; NUMBL; NUP85; PF4; PILRB; PTGDR2; PTPN6; PTPRC; PTPRF; PTPRN; RELA; RQCD1; SFTPD; SH2B2; SIGIRR; SOCS1; SOCS5; SPP1; STAT1; STAT4; STAT6; STAT5A; STAT5B; TLR2; TNFRSF1A; TNFSF11; TRAF6; Trbv13-2; TREM1; Zc3h15; ZNF675

Table S3. In GSE4107 (CRC) network, the most significant 100-subpathway information. The p-value is the significance of the subpathway based on the statistic S which was introduced in our previous method (PLOS ONE, 2014, 7(4): e31685).

<table>
<tr><th>KEGG</th><th>Title</th><th>Subpathway (linear signaling flow) with fold-change of the disease group over the control group (B <- A: A activates B; B |- A: A represses B)</th><th>The number of entries</th><th>P-value</th><th>S</th></tr>
<tr><td>hsa05215</td><td>Prostate cancer</td><td>TP53(1.310) |- EP300(0.739) |- CTNNB1(2.562) |- GSK3B(0.735) |- AKT1(1.297) <- PIK3CG(3.224) <- FGFR1(2.138) <- IGF1(2.529)</td><td>8</td><td>0.000017</td><td>100.432375</td></tr>
<tr><td>hsa05200</td><td>Pathways in cancer</td><td>MMP2(3.031) <- JUN(4.179) <- MAPK1(2.425) <- MAP2K1(1.162) <- ARAF(4.631) <- HRAS(1.027) <- SOS1(1.624) <- GRB2(1.613) <- IGF1R(2.299) <- IGF1(2.529)</td><td>10</td><td>0.000022</td><td>118.2492</td></tr>
<tr><td>hsa04512</td><td>ECM-receptor interaction</td><td>SDC2(3.091) <- TNC(9.557)</td><td>2</td><td>0.000026</td><td>33.692224</td></tr>
<tr><td>hsa05215</td><td>Prostate cancer</td><td>TP53(1.310) |- EP300(0.739) |- CREB5(3.206) |- GSK3B(0.735) |- AKT1(1.297) <- PIK3CG(3.224) <- FGFR1(2.138) <- IGF1(2.529)</td><td>8</td><td>0.000029</td><td>99.163054</td></tr>
<tr><td>hsa04340</td><td>Hedgehog signaling pathway</td><td>PTCH1(1.863) <- GLI2(2.878) |- CSNK1G1(0.587)</td><td>3</td><td>0.000035</td><td>38.234773</td></tr>
<tr><td>hsa05200</td><td>Pathways in cancer</td><td>MMP2(3.031) <- JUN(4.179) <- MAPK1(2.425) <- MAP2K1(1.162) <- ARAF(4.631) <- HRAS(1.027) <- SOS1(1.624) <- GRB2(1.613) <- FLT3(1.262) <- FLT3LG(3.022)</td><td>10</td><td>0.000046</td><td>111.5268</td></tr>
<tr><td>hsa04310</td><td>Wnt signaling pathway</td><td>PPARD(0.624) <- LEF1(0.551) <- EP300(0.739)</td><td>3</td><td>0.000047</td><td>38.018348</td></tr>
<tr><td>hsa05200</td><td>Pathways in cancer</td><td>MMP2(3.031) <- JUN(4.179) <- MAPK1(2.425) <- MAP2K1(1.162) <- ARAF(4.631) <- HRAS(1.027) <- SOS1(1.624) <- GRB2(1.613) <- FGFR1(2.138) <-</td><td>10</td><td>0.000051</td><td>120.6929</td></tr>
</table>

		FGF13(5.486)			
hsa04010	MAPK signaling pathway	ATF4(1.145) <- MAPKAPK2(1.586) <- MAPK12(1.277) <- MAP2K3(1.030) <- MAP3K6(2.707)	5	0.000057	58.361613
hsa05200	Pathways in cancer	CDK4(1.315) <- JUN(4.179) <- MAPK1(2.425) <- MAP2K1(1.162) <- ARAF(4.631) <- HRAS(1.027) <- SOS1(1.624) <- GRB2(1.613) <- IGF1R(2.299) <- IGF1(2.529)	10	0.000067	116.629834
hsa04310	Wnt signaling pathway	JUN(4.179) <- TCF7L1(2.735) <- CTNNB1(2.562) <- FBXW11(1.140)	4	0.000073	57.97362
hsa04510	Focal adhesion	CCND3(1.559) <- JUN(4.179) <- MAPK8(2.355) <- RAC2(2.502) <- VAV1(1.945) <- PIK3CG(3.224) <- HRAS(1.027) <- SOS1(1.624) <- GRB2(1.613) <- SHC3(1.838) <- FYN(4.286) <- CAV1(3.937) <- ITGB5(1.431)	13	0.000078	151.041913
hsa05200	Pathways in cancer	MMP2(3.031) <- MYC(3.052) <- MAPK1(2.425) <- MAP2K1(1.162) <- ARAF(4.631) <- HRAS(1.027) <- SOS1(1.624) <- GRB2(1.613) <- IGF1R(2.299) <- IGF1(2.529)	10	0.000082	109.631108
hsa05200	Pathways in cancer	FIGF(3.458) <- JUN(4.179) <- MAPK1(2.425) <- MAP2K1(1.162) <- ARAF(4.631) <- HRAS(1.027) <- SOS1(1.624) <- GRB2(1.613) <- IGF1R(2.299) <- IGF1(2.529)	10	0.000086	119.433355
hsa05200	Pathways in cancer	FIGF(3.458) <- JUN(4.179) <- MAPK1(2.425) <- MAP2K1(1.162) <- ARAF(4.631) <- HRAS(1.027) <- SOS1(1.624) <- GRB2(1.613) <- FGFR1(2.138) <- FGF13(5.486)	10	0.000088	121.877055
hsa05200	Pathways in cancer	CCND1(1.180) <- JUN(4.179) <- MAPK1(2.425) <- MAP2K1(1.162) <- ARAF(4.631) <- HRAS(1.027) <-	10	0.000088	116.286315

		SOS1(1.624) <- GRB2(1.613) <- IGF1R(2.299) <- IGF1(2.529)			
hsa04510	Focal adhesion	CCND3(1.559) <- CTNNB1(2.562) |- GSK3B(0.735) |- AKT1(1.297) <- ILK(1.467) <- PIK3CG(3.224) <- HRAS(1.027) <- SOS1(1.624) <- GRB2(1.613) <- SHC3(1.838) <- FYN(4.286) <- CAV1(3.937) <- ITGB5(1.431)	13	0.000104	144.056956
hsa04310	Wnt signaling pathway	JUN(4.179) <- TCF7L1(2.735) <- CTNNB1(2.562) |- CTNNBIP1(0.999)	4	0.000105	57.654029
hsa05200	Pathways in cancer	CCND1(1.180) <- JUN(4.179) <- MAPK1(2.425) <- MAP2K1(1.162) <- ARAF(4.631) <- HRAS(1.027) <- SOS1(1.624) <- GRB2(1.613) <- FGFR1(2.138) <- FGF13(5.486)	10	0.00011	118.730015
hsa05200	Pathways in cancer	CDK4(1.315) <- JUN(4.179) <- MAPK1(2.425) <- MAP2K1(1.162) <- ARAF(4.631) <- HRAS(1.027) <- SOS1(1.624) <- GRB2(1.613) <- FGFR1(2.138) <- FGF13(5.486)	10	0.000113	119.073534
hsa04310	Wnt signaling pathway	JUN(4.179) <- TCF7L1(2.735) <- CTNNB1(2.562) |- GSK3B(0.735) |- DVL3(1.608) <- FZD10(6.256) <- WNT3(3.147) <- PORCN(1.279)	8	0.000114	111.966934
hsa05200	Pathways in cancer	MMP2(3.031) <- FOS(36.201) <- MAPK1(2.425) <- MAP2K1(1.162) <- ARAF(4.631) <- HRAS(1.027) <- SOS1(1.624) <- GRB2(1.613) <- IGF1R(2.299) <- IGF1(2.529)	10	0.000114	127.428385
hsa04510	Focal adhesion	CCND3(1.559) <- CTNNB1(2.562) |- GSK3B(0.735) |- AKT1(1.297) <- ILK(1.467) <- PIK3CG(3.224) <- HRAS(1.027) <- SOS1(1.624) <- GRB2(1.613) <- SHC3(1.838) <- PDGFRB(2.851) <- IGF1(2.529)	12	0.000114	134.743081
hsa05200	Pathways in	MMP2(3.031) <- MYC(3.052) <- MAPK1(2.425) <-	10	0.000115	112.074808

<table>
<tr><td></td><td>cancer</td><td>MAP2K1(1.162) <- ARAF(4.631) <- HRAS(1.027) <- SOS1(1.624) <- GRB2(1.613) <- FGFR1(2.138) <- FGF13(5.486)</td><td></td><td></td><td></td></tr>
<tr><td>hsa04310</td><td>Wnt signaling pathway</td><td>JUN(4.179) <- TCF7L1(2.735) <- CTNNB1(2.562) <- PRKACA(1.130)</td><td>4</td><td>0.00012</td><td>57.74164</td></tr>
<tr><td>hsa04510</td><td>Focal adhesion</td><td>CCND3(1.559) <- JUN(4.179) <- MAPK8(2.355) <- RAC2(2.502) <- VAV1(1.945) <- PIK3CG(3.224) <- HRAS(1.027) <- SOS1(1.624) <- GRB2(1.613) <- SHC3(1.838) <- PDGFRB(2.851) <- IGF1(2.529)</td><td>12</td><td>0.00012</td><td>141.728038</td></tr>
<tr><td>hsa04512</td><td>ECM-receptor interaction</td><td>SDC2(3.091) <- FN1(5.594)</td><td>2</td><td>0.000125</td><td>32.846358</td></tr>
<tr><td>hsa04510</td><td>Focal adhesion</td><td>CCND3(1.559) <- JUN(4.179) <- MAPK8(2.355) <- RAC2(2.502) <- VAV1(1.945) <- PIK3CG(3.224) <- HRAS(1.027) <- SOS1(1.624) <- GRB2(1.613) <- SHC3(1.838) <- PTK2(2.151) <- PDGFRB(2.851) <- IGF1(2.529)</td><td>13</td><td>0.000127</td><td>150.313738</td></tr>
<tr><td>hsa05200</td><td>Pathways in cancer</td><td>MMP2(3.031) <- JUN(4.179) <- MAPK1(2.425) <- MAP2K1(1.162) <- ARAF(4.631) <- HRAS(1.027) <- SOS1(1.624) <- GRB2(1.613) <- PDGFRB(2.851) <- PDGFB(5.234)</td><td>10</td><td>0.00013</td><td>116.736026</td></tr>
<tr><td>hsa04510</td><td>Focal adhesion</td><td>CCND3(1.559) <- CTNNB1(2.562) |- GSK3B(0.735) |- AKT1(1.297) <- ILK(1.467) <- PIK3CG(3.224) <- HRAS(1.027) <- SOS1(1.624) <- GRB2(1.613) <- SHC3(1.838) <- PTK2(2.151) <- PDGFRB(2.851) <- IGF1(2.529)</td><td>13</td><td>0.00013</td><td>143.328781</td></tr>
<tr><td>hsa04510</td><td>Focal adhesion</td><td>CCND3(1.559) <- ELK1(2.593) <- MAPK1(2.425) <- MAP2K1(1.162) <- RAF1(1.813) <- PAK3(2.780) <- RAC2(2.502) <- VAV1(1.945) <- PIK3CG(3.224) <- HRAS(1.027) <- SOS1(1.624) <- GRB2(1.613) <-</td><td>16</td><td>0.000132</td><td>172.226929</td></tr>
</table>

		SHC3(1.838) <- FYN(4.286) <- CAV1(3.937) <- ITGB5(1.431)			
hsa04310	Wnt signaling pathway	JUN(4.179) <- TCF7L1(2.735) <- CTNNB1(2.562) |- GSK3B(0.735) |- DVL3(1.608) <- APC2(2.201) <- TBL1X(2.354)	7	0.000136	96.439788
hsa05200	Pathways in cancer	FIGF(3.458) <- JUN(4.179) <- MAPK1(2.425) <- MAP2K1(1.162) <- ARAF(4.631) <- HRAS(1.027) <- SOS1(1.624) <- GRB2(1.613) <- FLT3(1.262) <- FLT3LG(3.022)	10	0.000137	112.710955
hsa05200	Pathways in cancer	MMP2(3.031) <- FOS(36.201) <- MAPK1(2.425) <- MAP2K1(1.162) <- ARAF(4.631) <- HRAS(1.027) <- SOS1(1.624) <- GRB2(1.613) <- FGFR1(2.138) <- FGF13(5.486)	10	0.000139	129.872086
hsa04010	MAPK signaling pathway	ELK1(2.593) <- MAPK12(1.277) <- MAP2K3(1.030) <- MAP3K6(2.707)	4	0.000144	49.519989
hsa04010	MAPK signaling pathway	HSPB1(1.431) <- MAPKAPK2(1.586) <- MAPK12(1.277) <- MAP2K3(1.030) <- MAP3K6(2.707)	5	0.000145	59.544902
hsa05215	Prostate cancer	CCND1(1.180) <- TCF7L1(2.735) <- CTNNB1(2.562) |- GSK3B(0.735) |- AKT1(1.297) <- PIK3CG(3.224) <- FGFR1(2.138) <- IGF1(2.529)	8	0.000146	100.872409
hsa05200	Pathways in cancer	MMP2(3.031) <- JUN(4.179) <- MAPK1(2.425) <- MAP2K1(1.162) <- ARAF(4.631) <- HRAS(1.027) <- RET(2.212)	7	0.000147	82.11207
hsa05200	Pathways in cancer	MMP2(3.031) <- JUN(4.179) <- MAPK1(2.425) <- MAP2K1(1.162) <- ARAF(4.631) <- HRAS(1.027) <- SOS1(1.624) <- GRB2(1.613) <- BCR(1.241)	9	0.000148	99.976292
hsa04310	Wnt signaling	JUN(4.179) <- TCF7L1(2.735) <- CTNNB1(2.562) |-	8	0.000156	114.2165

<table>
<tr><td></td><td>pathway</td><td>GSK3B(0.735) |- DVL3(1.608) <- FZD10(6.256) <- WNT3(3.147) |- WIF1(0.661)</td><td></td><td></td><td></td></tr>
<tr><td>hsa05200</td><td>Pathways in cancer</td><td>MMP2(3.031) <- ETS1(1.805) <- MAPK1(2.425) <- MAP2K1(1.162) <- ARAF(4.631) <- HRAS(1.027) <- SOS1(1.624) <- GRB2(1.613) <- IGF1R(2.299) <- IGF1(2.529)</td><td>10</td><td>0.000157</td><td>106.505111</td></tr>
<tr><td>hsa04310</td><td>Wnt signaling pathway</td><td>JUN(4.179) <- TCF7L1(2.735) <- CTNNB1(2.562) |- GSK3B(0.735) |- DVL3(1.608) <- APC2(2.201) <- AXIN2(2.307) <- CSNK1A1(1.963)</td><td>8</td><td>0.00016</td><td>103.109526</td></tr>
<tr><td>hsa05200</td><td>Pathways in cancer</td><td>MMP2(3.031) <- ETS1(1.805) <- MAPK1(2.425) <- MAP2K1(1.162) <- ARAF(4.631) <- HRAS(1.027) <- SOS1(1.624) <- GRB2(1.613) <- FGFR1(2.138) <- FGF13(5.486)</td><td>10</td><td>0.000161</td><td>108.948811</td></tr>
<tr><td>hsa04510</td><td>Focal adhesion</td><td>CCND3(1.559) <- ELK1(2.593) <- MAPK1(2.425) <- MAP2K1(1.162) <- RAF1(1.813) <- PAK3(2.780) <- RAC2(2.502) <- VAV1(1.945) <- PIK3CG(3.224) <- HRAS(1.027) <- SOS1(1.624) <- GRB2(1.613) <- SHC3(1.838) <- PDGFRB(2.851) <- IGF1(2.529)</td><td>15</td><td>0.000161</td><td>162.445318</td></tr>
<tr><td>hsa04510</td><td>Focal adhesion</td><td>BIRC3(2.201) <- ELK1(2.593) <- MAPK1(2.425) <- MAP2K1(1.162) <- RAF1(1.813) <- PAK3(2.780) <- RAC2(2.502) <- VAV1(1.945) <- PIK3CG(3.224) <- HRAS(1.027) <- SOS1(1.624) <- GRB2(1.613) <- SHC3(1.838) <- FYN(4.286) <- CAV1(3.937) <- ITGB5(1.431)</td><td>16</td><td>0.000163</td><td>171.355023</td></tr>
<tr><td>hsa04310</td><td>Wnt signaling pathway</td><td>JUN(4.179) <- TCF7L1(2.735) <- CTNNB1(2.562) <- TBL1X(2.354)</td><td>4</td><td>0.000167</td><td>60.77765</td></tr>
<tr><td>hsa04510</td><td>Focal adhesion</td><td>CCND3(1.559) <- ELK1(2.593) <- MAPK1(2.425) <- MAP2K1(1.162) <- RAF1(1.813) <- PAK3(2.780) <- RAC2(2.502) <- VAV1(1.945) <- PIK3CG(3.224) <-</td><td>16</td><td>0.000168</td><td>171.498754</td></tr>
</table>

		HRAS(1.027) <- SOS1(1.624) <- GRB2(1.613) <- SHC3(1.838) <- PTK2(2.151) <- PDGFRB(2.851) <- IGF1(2.529)			
hsa04510	Focal adhesion	CCND3(1.559) <- JUN(4.179) <- MAPK8(2.355) <- RAC2(2.502) <- VAV1(1.945) <- PIK3CG(3.224) <- HRAS(1.027) <- SOS1(1.624) <- GRB2(1.613) <- SHC3(1.838) <- PTK2(2.151) <- PRKCA(3.061) <- ITGB5(1.431)	13	0.000169	142.835253
hsa04510	Focal adhesion	CCND3(1.559) <- CTNNB1(2.562) |- GSK3B(0.735) |- AKT1(1.297) <- ILK(1.467) <- PIK3CG(3.224) <- HRAS(1.027) <- SOS1(1.624) <- GRB2(1.613) <- SHC3(1.838) <- PTK2(2.151) <- PRKCA(3.061) <- ITGB5(1.431)	13	0.000169	135.850296
hsa04510	Focal adhesion	CCND3(1.559) <- CTNNB1(2.562) |- GSK3B(0.735) |- AKT1(1.297) <- PIK3CG(3.224) <- HRAS(1.027) <- SOS1(1.624) <- GRB2(1.613) <- SHC3(1.838) <- PDGFRB(2.851) <- IGF1(2.529)	11	0.000171	123.449184
hsa04510	Focal adhesion	CCND3(1.559) <- ELK1(2.593) <- MAPK1(2.425) <- MAP2K1(1.162) <- RAF1(1.813) <- HRAS(1.027) <- SOS1(1.624) <- GRB2(1.613) <- SHC3(1.838) <- PDGFRB(2.851) <- IGF1(2.529)	11	0.000172	121.208394
hsa04510	Focal adhesion	CCND3(1.559) <- ELK1(2.593) <- MAPK1(2.425) <- MAP2K1(1.162) <- PAK3(2.780) <- RAC2(2.502) <- VAV1(1.945) <- PIK3CG(3.224) <- HRAS(1.027) <- SOS1(1.624) <- GRB2(1.613) <- SHC3(1.838) <- FYN(4.286) <- CAV1(3.937) <- ITGB5(1.431)	15	0.000176	162.831731
hsa04510	Focal adhesion	BIRC3(2.201) <- ELK1(2.593) <- MAPK1(2.425) <- MAP2K1(1.162) <- RAF1(1.813) <- PAK3(2.780) <- RAC2(2.502) <- VAV1(1.945) <- PIK3CG(3.224) <-	15	0.000187	161.573412

		HRAS(1.027) <- SOS1(1.624) <- GRB2(1.613) <- SHC3(1.838) <- PDGFRB(2.851) <- IGF1(2.529)			
hsa04510	Focal adhesion	CCND3(1.559) <- ELK1(2.593) <- MAPK1(2.425) <- MAP2K1(1.162) <- PAK3(2.780) <- RAC2(2.502) <- VAV1(1.945) <- PIK3CG(3.224) <- HRAS(1.027) <- SOS1(1.624) <- GRB2(1.613) <- SHC3(1.838) <- PDGFRB(2.851) <- IGF1(2.529)	14	0.00019	153.394963
hsa05200	Pathways in cancer	MMP2(3.031) <- FOS(36.201) <- MAPK1(2.425) <- MAP2K1(1.162) <- ARAF(4.631) <- HRAS(1.027) <- SOS1(1.624) <- GRB2(1.613) <- FLT3(1.262) <- FLT3LG(3.022)	10	0.000193	120.705985
hsa04510	Focal adhesion	CCND3(1.559) <- ELK1(2.593) <- MAPK1(2.425) <- MAP2K1(1.162) <- RAF1(1.813) <- HRAS(1.027) <- SOS1(1.624) <- GRB2(1.613) <- SHC3(1.838) <- PTK2(2.151) <- PDGFRB(2.851) <- IGF1(2.529)	12	0.000194	129.741055
hsa04510	Focal adhesion	BIRC3(2.201) <- ELK1(2.593) <- MAPK1(2.425) <- MAP2K1(1.162) <- RAF1(1.813) <- HRAS(1.027) <- SOS1(1.624) <- GRB2(1.613) <- SHC3(1.838) <- PDGFRB(2.851) <- IGF1(2.529)	11	0.000195	120.336487
hsa04310	Wnt signaling pathway	MMP7(1.088) <- TCF7L1(2.735) <- CTNNB1(2.562) |- GSK3B(0.735) |- DVL3(1.608) <- FZD10(6.256) <- WNT3(3.147) <- PORCN(1.279)	8	0.000196	99.88631
hsa04310	Wnt signaling pathway	JUN(4.179) <- TCF7L1(2.735) <- CTNNB1(2.562) |- GSK3B(0.735) |- DVL3(1.608) <- FZD10(6.256) <- WNT3(3.147)	7	0.000197	103.367927
hsa04510	Focal adhesion	BIRC3(2.201) <- ELK1(2.593) <- MAPK1(2.425) <- MAP2K1(1.162) <- RAF1(1.813) <- PAK3(2.780) <- RAC2(2.502) <- VAV1(1.945) <- PIK3CG(3.224) <- HRAS(1.027) <- SOS1(1.624) <- GRB2(1.613) <-	16	0.000197	170.626848

<table>
<tr><td></td><td></td><td>SHC3(1.838) <- PTK2(2.151) <- PDGFRB(2.851) <- IGF1(2.529)</td><td></td><td></td><td></td></tr>
<tr><td>hsa03320</td><td>PPAR signaling pathway</td><td>HMGCS2(0.162) <- RXRB(0.447)</td><td>2</td><td>0.000198</td><td>36.398441</td></tr>
<tr><td>hsa04916</td><td>Melanogenesis</td><td>MITF(2.570) <- TCF7L1(2.735) <- CTNNB1(2.562) |- GSK3B(0.735) |- DVL3(1.608) <- GNAO1(7.981) <- FZD10(6.256) <- WNT3(3.147)</td><td>8</td><td>0.000199</td><td>113.101926</td></tr>
<tr><td>hsa04510</td><td>Focal adhesion</td><td>CCND3(1.559) <- CTNNB1(2.562) |- GSK3B(0.735) |- AKT1(1.297) <- PIK3CG(3.224) <- HRAS(1.027) <- SOS1(1.624) <- GRB2(1.613) <- SHC3(1.838) <- PTK2(2.151) <- PDGFRB(2.851) <- IGF1(2.529)</td><td>12</td><td>0.000199</td><td>131.981845</td></tr>
<tr><td>hsa05200</td><td>Pathways in cancer</td><td>MMP2(3.031) <- MYC(3.052) <- MAPK1(2.425) <- MAP2K1(1.162) <- ARAF(4.631) <- HRAS(1.027) <- SOS1(1.624) <- GRB2(1.613) <- FLT3(1.262) <- FLT3LG(3.022)</td><td>10</td><td>0.000203</td><td>102.908708</td></tr>
<tr><td>hsa04722</td><td>Neurotrophin signaling pathway</td><td>ATF4(1.145) <- RPS6KA2(1.936) <- MAPK1(2.425) <- MAP2K1(1.162) <- RAF1(1.813) <- HRAS(1.027) <- SOS1(1.624) <- GRB2(1.613) <- SH2B3(1.476) <- NTRK3(2.746) <- NTF3(3.162)</td><td>11</td><td>0.000203</td><td>114.014919</td></tr>
<tr><td>hsa04510</td><td>Focal adhesion</td><td>BIRC3(2.201) <- ELK1(2.593) <- MAPK1(2.425) <- MAP2K1(1.162) <- RAF1(1.813) <- HRAS(1.027) <- SOS1(1.624) <- GRB2(1.613) <- SHC3(1.838) <- PTK2(2.151) <- PDGFRB(2.851) <- IGF1(2.529)</td><td>12</td><td>0.000205</td><td>128.869148</td></tr>
<tr><td>hsa04310</td><td>Wnt signaling pathway</td><td>JUN(4.179) <- TCF7L1(2.735) |- CTBP2(0.949)</td><td>3</td><td>0.000206</td><td>46.207104</td></tr>
<tr><td>hsa04510</td><td>Focal adhesion</td><td>CCND3(1.559) <- JUN(4.179) <- MAPK8(2.355) <- RAC2(2.502) <- VAV1(1.945) <- PIK3CG(3.224) <- HRAS(1.027) <- SOS1(1.624) <- GRB2(1.613) <-</td><td>12</td><td>0.000207</td><td>132.489748</td></tr>
</table>

		SHC3(1.838) <- PTK2(2.151) <- ITGB5(1.431)			
hsa05200	Pathways in cancer	IL8(4.276) <- JUN(4.179) <- MAPK1(2.425) <- MAP2K1(1.162) <- ARAF(4.631) <- HRAS(1.027) <- SOS1(1.624) <- GRB2(1.613) <- IGF1R(2.299) <- IGF1(2.529)	10	0.000211	117.318822
hsa04510	Focal adhesion	BIRC3(2.201) <- ELK1(2.593) <- MAPK1(2.425) <- MAP2K1(1.162) <- PAK3(2.780) <- RAC2(2.502) <- VAV1(1.945) <- PIK3CG(3.224) <- HRAS(1.027) <- SOS1(1.624) <- GRB2(1.613) <- SHC3(1.838) <- PDGFRB(2.851) <- IGF1(2.529)	14	0.000212	152.523056
hsa04510	Focal adhesion	CCND3(1.559) <- ELK1(2.593) <- MAPK1(2.425) <- MAP2K1(1.162) <- PAK3(2.780) <- RAC2(2.502) <- VAV1(1.945) <- PIK3CG(3.224) <- HRAS(1.027) <- SOS1(1.624) <- GRB2(1.613) <- SHC3(1.838) <- PTK2(2.151) <- PDGFRB(2.851) <- IGF1(2.529)	15	0.000217	162.103555
hsa04010	MAPK signaling pathway	HSPB1(1.431) <- MAPKAPK2(1.586) <- MAPK12(1.277) <- MAP2K3(1.030) <- MAP3K5(1.143) <- DAXX(1.204) <- TGFBR2(1.728) <- TGFB2(2.927)	8	0.00022	85.243979
hsa04340	Hedgehog signaling pathway	WNT3(3.147) <- GLI2(2.878) |- CSNK1G1(0.587)	3	0.000223	38.776013
hsa04510	Focal adhesion	CCND3(1.559) <- CTNNB1(2.562) |- GSK3B(0.735) |- AKT1(1.297) <- PIK3CG(3.224) <- HRAS(1.027) <- SOS1(1.624) <- GRB2(1.613) <- SHC3(1.838) <- FYN(4.286) <- CAV1(3.937) <- ITGB5(1.431)	12	0.000224	132.710021
hsa05200	Pathways in cancer	FIGF(3.458) <- FOS(36.201) <- MAPK1(2.425) <- MAP2K1(1.162) <- ARAF(4.631) <- HRAS(1.027) <- SOS1(1.624) <- GRB2(1.613) <- IGF1R(2.299) <-	10	0.000229	128.635013

		IGF1(2.529)			
hsa04722	Neurotrophin signaling pathway	RELA(1.663) <- NFKBIA(1.558) <- AKT1(1.297) <- PIK3CG(3.224) <- HRAS(1.027) <- SOS1(1.624) <- GRB2(1.613) <- SH2B3(1.476) <- NTRK3(2.746) <- NTF3(3.162)	10	0.00023	104.568524
hsa04510	Focal adhesion	BIRC3(2.201) <- ELK1(2.593) <- MAPK1(2.425) <- MAP2K1(1.162) <- PAK3(2.780) <- RAC2(2.502) <- VAV1(1.945) <- PIK3CG(3.224) <- HRAS(1.027) <- SOS1(1.624) <- GRB2(1.613) <- SHC3(1.838) <- FYN(4.286) <- CAV1(3.937) <- ITGB5(1.431)	15	0.00023	161.959824
hsa04510	Focal adhesion	CCND3(1.559) <- CTNNB1(2.562) |- GSK3B(0.735) |- AKT1(1.297) <- ILK(1.467) <- PIK3CG(3.224) <- HRAS(1.027) <- SOS1(1.624) <- GRB2(1.613) <- SHC3(1.838) <- PTK2(2.151) <- ITGB5(1.431)	12	0.000231	125.504791
hsa04510	Focal adhesion	BIRC3(2.201) <- ELK1(2.593) <- MAPK1(2.425) <- MAP2K1(1.162) <- PAK3(2.780) <- RAC2(2.502) <- VAV1(1.945) <- PIK3CG(3.224) <- HRAS(1.027) <- SOS1(1.624) <- GRB2(1.613) <- SHC3(1.838) <- PTK2(2.151) <- PDGFRB(2.851) <- IGF1(2.529)	15	0.000232	161.231649
hsa04510	Focal adhesion	CCND3(1.559) <- ELK1(2.593) <- MAPK1(2.425) <- MAP2K1(1.162) <- RAF1(1.813) <- HRAS(1.027) <- SOS1(1.624) <- GRB2(1.613) <- SHC3(1.838) <- FYN(4.286) <- CAV1(3.937) <- ITGB5(1.431)	12	0.000234	130.46923
hsa04310	Wnt signaling pathway	JUN(4.179) <- TCF7L1(2.735) <- CTNNB1(2.562) <- APC2(2.201) <- TBL1X(2.354)	5	0.000247	72.162795
hsa04310	Wnt signaling pathway	JUN(4.179) <- TCF7L1(2.735) <- CTNNB1(2.562) |- GSK3B(0.735) |- DVL3(1.608) <- AXIN2(2.307) <- CSNK1A1(1.963)	7	0.000253	91.970774
hsa05200	Pathways in	FIGF(3.458) <- JUN(4.179) <- MAPK1(2.425) <-	10	0.000254	117.920181

	cancer	MAP2K1(1.162) <- ARAF(4.631) <- HRAS(1.027) <- SOS1(1.624) <- GRB2(1.613) <- PDGFRB(2.851) <- PDGFB(5.234)			
hsa04310	Wnt signaling pathway	JUN(4.179) <- TCF7L1(2.735) <- CTNNB1(2.562) <- RUVBL1(1.462)	4	0.000255	58.602051
hsa05200	Pathways in cancer	IL8(4.276) <- JUN(4.179) <- MAPK1(2.425) <- MAP2K1(1.162) <- ARAF(4.631) <- HRAS(1.027) <- SOS1(1.624) <- GRB2(1.613) <- FGFR1(2.138) <- FGF13(5.486)	10	0.000257	119.762522
hsa05200	Pathways in cancer	MMP2(3.031) <- JUN(4.179) <- MAPK1(2.425) <- MAP2K1(1.162) <- ARAF(4.631) <- HRAS(1.027) <- NTRK1(1.225)	7	0.000261	81.150701
hsa05200	Pathways in cancer	FIGF(3.458) <- MYC(3.052) <- MAPK1(2.425) <- MAP2K1(1.162) <- ARAF(4.631) <- HRAS(1.027) <- SOS1(1.624) <- GRB2(1.613) <- FGFR1(2.138) <- FGF13(5.486)	10	0.000269	113.303414
hsa05200	Pathways in cancer	CCND1(1.180) <- JUN(4.179) <- MAPK1(2.425) |- DAPK1(0.438)	4	0.000271	50.453133
hsa05200	Pathways in cancer	CDK4(1.315) <- JUN(4.179) <- MAPK1(2.425) <- MAP2K1(1.162) <- ARAF(4.631) <- HRAS(1.027) <- SOS1(1.624) <- GRB2(1.613) <- FLT3(1.262) <- FLT3LG(3.022)	10	0.000275	109.907434
hsa04510	Focal adhesion	CCND3(1.559) <- JUN(4.179) <- MAPK8(2.355) <- RAC2(2.502) <- VAV1(1.945) <- PIK3CG(3.224) <- HRAS(1.027) <- SOS1(1.624) <- GRB2(1.613) <- SHC3(1.838) <- PTK2(2.151) |- PTEN(0.599)	12	0.000275	129.820569
hsa04510	Focal adhesion	BIRC3(2.201) <- ELK1(2.593) <- MAPK1(2.425) <- MAP2K1(1.162) <- RAF1(1.813) <- HRAS(1.027) <- SOS1(1.624) <- GRB2(1.613) <- SHC3(1.838) <-	12	0.000275	129.597323

		FYN(4.286) <- CAV1(3.937) <- ITGB5(1.431)			
hsa04510	Focal adhesion	CCND3(1.559) <- ELK1(2.593) <- MAPK1(2.425) <- MAP2K1(1.162) <- RAF1(1.813) <- PAK3(2.780) <- RAC2(2.502) <- VAV1(1.945) <- PIK3CG(3.224) <- HRAS(1.027) <- SOS1(1.624) <- GRB2(1.613) <- SHC3(1.838) <- PTK2(2.151) <- PRKCA(3.061) <- ITGB5(1.431)	16	0.000279	164.020269
hsa05200	Pathways in cancer	CCND1(1.180) <- JUN(4.179) <- MAPK1(2.425) <- MAP2K1(1.162) <- ARAF(4.631) <- HRAS(1.027) <- SOS1(1.624) <- GRB2(1.613) <- FLT3(1.262) <- FLT3LG(3.022)	10	0.00028	109.563915
hsa05020	Prion diseases	NOTCH1(1.633) <- PRNP(2.541)	2	0.000281	25.726411
hsa05200	Pathways in cancer	CDK4(1.315) <- FOS(36.201) <- MAPK1(2.425) <- MAP2K1(1.162) <- ARAF(4.631) <- HRAS(1.027) <- SOS1(1.624) <- GRB2(1.613) <- IGF1R(2.299) <- IGF1(2.529)	10	0.000282	125.936852
hsa04510	Focal adhesion	CCND3(1.559) <- CTNNB1(2.562) |- GSK3B(0.735) |- AKT1(1.297) <- PIK3CG(3.224) <- HRAS(1.027) <- SOS1(1.624) <- GRB2(1.613) <- SHC3(1.838) <- PTK2(2.151) <- PRKCA(3.061) <- ITGB5(1.431)	12	0.000289	124.50336
hsa05200	Pathways in cancer	FIGF(3.458) <- FOS(36.201) <- MAPK1(2.425) <- MAP2K1(1.162) <- ARAF(4.631) <- HRAS(1.027) <- SOS1(1.624) <- GRB2(1.613) <- FGFR1(2.138) <- FGF13(5.486)	10	0.000292	131.078713
hsa05200	Pathways in cancer	FIGF(3.458) <- MYC(3.052) <- MAPK1(2.425) <- MAP2K1(1.162) <- ARAF(4.631) <- HRAS(1.027) <- SOS1(1.624) <- GRB2(1.613) <- IGF1R(2.299) <- IGF1(2.529)	10	0.000298	110.859714

hsa04115	p53 signaling pathway	CDK6(0.141) |- CDKN1A(1.707) <- TP53(1.310) <- ATM(1.918)	4	0.000298	56.447222
hsa04722	Neurotrophin signaling pathway	ATF4(1.145) <- RPS6KA2(1.936) <- MAPK1(2.425) <- MAP2K1(1.162) <- RAF1(1.813) <- HRAS(1.027) <- SOS1(1.624) <- GRB2(1.613) <- SH2B3(1.476) <- NTRK1(1.225) <- NTF3(3.162)	11	0.000301	113.914276

Table S4. In GSE1297 (AD) network, the most significant 100-subpathway information. The p-value is the significance of the subpathway based on the statistic S which was introduced in our previous method (PLOS ONE, 2014, 7(4): e31685).

**KEGG**	**Title**	**Subpathway (linear signaling flow) with fold-change of the disease group over the control group** (B <- A: A activates B; B |- A: A represses B)	**The number of entries**	**P-value**	**S**
hsa04510	Focal adhesion	ITGB3(5.450) <- PXN(1.203) <- ILK(1.014) <- PIK3R1(1.231) <- PTK2(1.059) <- FLT4(1.807) <- HGF(2.375)	7	0	83.534628
hsa04510	Focal adhesion	ITGB3(5.450) <- PXN(1.203) <- ILK(1.014) <- PIK3R1(1.231) <- FLT4(1.807) <- HGF(2.375)	6	0	75.073347
hsa04310	Wnt signaling pathway	CSNK2A2(1.127) <- DVL2(1.888) <- FZD5(1.630) <- WNT6(2.014) |- WIF1(0.342)	5	0.0001	72.793857
hsa04510	Focal adhesion	ITGB3(5.450) <- PXN(1.203) <- ILK(1.014) <- PIK3R1(1.231) <- PTK2(1.059) <- PRKCA(1.452) <- ITGB3(5.450)	7	0.0001	89.816607
hsa04510	Focal adhesion	BAD(1.180) |- MAP2K1(0.692) <- RAF1(0.998) <- PAK3(0.732) <- RAC1(0.943) <- VAV2(0.631) <- PIK3CA(0.633) <- HRAS(0.806) <- SOS2(0.729) <- GRB2(0.940) <- SHC3(0.873)	11	0.0002	116.991142
hsa04510	Focal adhesion	ITGB3(5.450) <- PXN(1.203) <- ILK(1.014) <- PIK3R1(1.231) <- PTK2(1.059) <- ITGB3(5.450)	6	0.0002	79.556872
hsa04510	Focal adhesion	BAD(1.180) |- MAP2K1(0.692) <- RAF1(0.998) <- PAK3(0.732) <- RAC1(0.943) <- VAV2(0.631) <- PIK3CA(0.633) <- HRAS(0.806) <- SOS2(0.729) <- GRB2(0.940) <- SHC3(0.873) <- MET(0.516)	12	0.0003	130.02502
hsa04062	Chemokine signaling pathway	RELA(1.165) <- NFKBIB(1.228) <- IKBKB(2.069) <- AKT3(1.232) <- PIK3R1(1.231) <- CSK(1.464) <- GNAI2(1.209) <- CXCR3(1.690) <- CXCL5(4.090)	9	0.0003	102.652236
hsa04010	MAPK signaling pathway	FGFR4(1.600) <- FGF20(4.313)	2	0.0004	35.328107
hsa04510	Focal adhesion	BAD(1.180) |- RAF1(0.998) <- PAK3(0.732) <- RAC1(0.943) <- VAV2(0.631) <- PIK3CA(0.633) <- HRAS(0.806) <- SOS2(0.729) <- GRB2(0.940) <-	11	0.0004	119.744174

		SHC3(0.873) <- MET(0.516)			
hsa04510	Focal adhesion	BAD(1.180) |- MAP2K1(0.692) <- RAF1(0.998) <- PAK3(0.732) <- RAC1(0.943) <- VAV2(0.631) <- PIK3CA(0.633) <- HRAS(0.806) <- SOS2(0.729) <- GRB2(0.940) <- SHC3(0.873)	11	0.0004	116.991142
hsa04510	Focal adhesion	BAD(1.180) |- MAP2K1(0.692) <- RAF1(0.998) <- PAK3(0.732) <- RAC1(0.943) <- VAV2(0.631) <- PIK3CA(0.633) <- HRAS(0.806) <- SOS2(0.729) <- GRB2(0.940) <- SHC3(0.873)	11	0.0004	116.991142
hsa04510	Focal adhesion	BAD(1.180) |- MAP2K1(0.692) <- PAK3(0.732) <- RAC1(0.943) <- VAV2(0.631) <- PIK3CA(0.633) <- HRAS(0.806) <- SOS2(0.729) <- GRB2(0.940) <- SHC3(0.873) <- MET(0.516)	11	0.0005	121.622575
hsa04062	Chemokine signaling pathway	MAPK1(0.731) <- MAP2K1(0.692) <- RAF1(0.998) <- HRAS(0.806) <- PIK3CA(0.633) <- GNB5(0.424) <- GNAI1(0.803) <- CCR6(0.793) <- CXCL3(0.876)	9	0.0005	97.95825
hsa04722	Neurotrophin signaling pathway	ATF4(0.991) <- RPS6KA3(0.945) <- MAPK1(0.731) <- MAP2K1(0.692) <- RAF1(0.998) <- HRAS(0.806) <- SOS2(0.729) <- GRB2(0.940) <- SHC3(0.873) <- NTRK2(0.632)	10	0.0006	100.297514
hsa04510	Focal adhesion	BAD(1.180) |- MAP2K1(0.692) <- RAF1(0.998) <- PAK3(0.732) <- RAC1(0.943) <- VAV2(0.631) <- PIK3CA(0.633) <- HRAS(0.806) <- SOS2(0.729) <- GRB2(0.940) <- SHC3(0.873)	11	0.0006	116.991142
hsa04062	Chemokine signaling pathway	RELA(1.165) <- NFKBIB(1.228) <- IKBKB(2.069) <- AKT3(1.232) <- PIK3R1(1.231) <- CSK(1.464) <- GNAI2(1.209) <- CXCR3(1.690) <- GRK4(1.645)	9	0.0006	94.84064
hsa04062	Chemokine signaling pathway	GSK3A(0.891) |- AKT3(1.232) <- PIK3R1(1.231) <- CSK(1.464) <- GNAI2(1.209) <- CXCR3(1.690) <- CXCL5(4.090)	7	0.0006	81.453514
hsa04062	Chemokine signaling pathway	ROCK2(1.508) <- RHOA(1.025) <- VAV1(1.126) <- ITK(1.131) <- PIK3R1(1.231) <- CSK(1.464) <- GNAI2(1.209) <- CXCR3(1.690) <- CXCL5(4.090)	9	0.0007	97.298392

hsa04510	Focal adhesion	BAD(1.180) |- MAP2K1(0.692) <- RAF1(0.998) <- PAK3(0.732) <- RAC1(0.943) <- VAV2(0.631) <- PIK3CA(0.633) <- HRAS(0.806) <- SOS2(0.729) <- GRB2(0.940) <- SHC3(0.873)	11	0.0008	116.991142
hsa04062	Chemokine signaling pathway	RELA(1.165) <- NFKBIB(1.228) <- IKBKB(2.069) <- AKT3(1.232) <- PIK3R1(1.231) <- CSK(1.464) <- GNAI2(1.209) <- CXCR3(1.690) <- ARRB2(1.084)	9	0.0008	94.524707
hsa04360	Axon guidance	PAK3(0.732) <- RAC1(0.943) |- PLXNB3(1.627) <- SEMA4C(1.283)	4	0.0008	48.132661
hsa05222	Small cell lung cancer	TRAF2(1.956) <- RELA(1.165) <- NFKBIA(1.386) <- IKBKB(2.069) <- AKT3(1.232) <- PIK3R1(1.231) <- PTK2(1.059) <- ITGA3(2.104) <- LAMA4(1.588)	9	0.0009	94.951153
hsa05222	Small cell lung cancer	BIRC2(1.016) <- RELA(1.165) <- NFKBIA(1.386) <- IKBKB(2.069) <- AKT3(1.232) <- PIK3R1(1.231) <- PTK2(1.059) <- ITGA3(2.104) <- LAMA4(1.588)	9	0.001	91.683159
hsa04510	Focal adhesion	BAD(1.180) |- RAF1(0.998) <- PAK3(0.732) <- RAC1(0.943) <- VAV2(0.631) <- PIK3CA(0.633) <- HRAS(0.806) <- SOS2(0.729) <- GRB2(0.940) <- SHC3(0.873)	10	0.001	106.693423
hsa04062	Chemokine signaling pathway	PXN(1.203) <- PTK2(1.059) <- PIK3R1(1.231) <- CSK(1.464) <- GNAI2(1.209) <- CXCR3(1.690) <- CXCL5(4.090)	7	0.001	80.986849
hsa04722	Neurotrophin signaling pathway	ATF4(0.991) <- RPS6KA3(0.945) <- MAPK1(0.731) <- MAP2K1(0.692) <- RAF1(0.998) <- HRAS(0.806) <- SOS2(0.729) <- GRB2(0.940) <- SHC3(0.873) <- NTRK2(0.632)	10	0.0011	100.297514
hsa04510	Focal adhesion	BAD(1.180) |- MAP2K1(0.692) <- PAK3(0.732) <- RAC1(0.943) <- VAV2(0.631) <- PIK3CA(0.633) <- HRAS(0.806) <- SOS2(0.729) <- GRB2(0.940) <- SHC3(0.873)	10	0.0011	108.571824
hsa04510	Focal adhesion	BAD(1.180) |- RAF1(0.998) <- PAK3(0.732) <- RAC1(0.943) <- VAV2(0.631) <- PIK3CA(0.633) <- HRAS(0.806) <- SOS2(0.729) <- GRB2(0.940) <- SHC3(0.873)	10	0.0011	106.693423

hsa05218	Melanoma	CCND1(0.798) <- MAPK1(0.731) <- MAP2K1(0.692) <- RAF1(0.998) <- HRAS(0.806) <- MET(0.516) <- FGF22(0.732)	7	0.0012	76.568936
hsa05222	Small cell lung cancer	BCL2L1(1.567) <- RELA(1.165) <- NFKBIA(1.386) <- IKBKB(2.069) <- AKT3(1.232) <- PIK3R1(1.231) <- PTK2(1.059) <- ITGA3(2.104) <- LAMA4(1.588)	9	0.0013	92.487954
hsa04510	Focal adhesion	BAD(1.180) |- RAF1(0.998) <- PAK3(0.732) <- RAC1(0.943) <- VAV2(0.631) <- PIK3CA(0.633) <- HRAS(0.806) <- SOS2(0.729) <- GRB2(0.940) <- SHC3(0.873)	10	0.0013	106.693423
hsa04510	Focal adhesion	BAD(1.180) |- RAF1(0.998) <- PAK3(0.732) <- RAC1(0.943) <- VAV2(0.631) <- PIK3CA(0.633) <- HRAS(0.806) <- SOS2(0.729) <- GRB2(0.940) <- SHC3(0.873)	10	0.0013	106.693423
hsa04722	Neurotrophin signaling pathway	ATF4(0.991) <- RPS6KA3(0.945) <- MAPK1(0.731) <- MAP2K1(0.692) <- RAF1(0.998) <- HRAS(0.806) <- SOS2(0.729) <- GRB2(0.940) <- SHC3(0.873)	9	0.0014	90.196122
hsa04010	MAPK signaling pathway	ATF4(0.991) <- RPS6KA3(0.945) <- MAPK1(0.731) <- MAP2K1(0.692) <- MAP3K1(0.999) <- RAC1(0.943)	6	0.0014	61.844431
hsa04910	Insulin signaling pathway	RHOQ(1.970) <- TRIP10(1.312)	2	0.0014	25.285305
hsa04510	Focal adhesion	BAD(1.180) |- MAP2K1(0.692) <- PAK3(0.732) <- RAC1(0.943) <- VAV2(0.631) <- PIK3CA(0.633) <- HRAS(0.806) <- SOS2(0.729) <- GRB2(0.940) <- SHC3(0.873)	10	0.0014	108.571824
hsa04062	Chemokine signaling pathway	MAPK1(0.731) <- MAP2K1(0.692) <- RAF1(0.998) <- HRAS(0.806) <- PIK3CA(0.633) <- GNB5(0.424) <- GNAI1(0.803) <- CCR6(0.793) <- ARRB1(0.962)	9	0.0014	94.79842
hsa04510	Focal adhesion	BAD(1.180) |- RAF1(0.998) <- PAK3(0.732) <- RAC1(0.943) <- VAV2(0.631) <- PIK3CA(0.633) <- HRAS(0.806) <- SOS2(0.729) <- GRB2(0.940) <- SHC3(0.873)	10	0.0015	106.693423

hsa04062	Chemokine signaling pathway	ROCK2(1.508) <- RHOA(1.025) <- VAV1(1.126) <- ITK(1.131) <- CSK(1.464) <- GNAI2(1.209) <- CXCR3(1.690) <- CXCL5(4.090)	8	0.0015	85.500397
hsa04360	Axon guidance	MAPK3(1.278) <- NRAS(1.214) |- RASA1(0.927) <- EPHA3(0.283) <- EFNA5(0.460)	5	0.0015	65.273243
hsa05014	Amyotrophic lateral sclerosis (ALS)	NEFL(0.383) <- PRPH(0.871)	2	0.0016	33.004948
hsa04510	Focal adhesion	BAD(1.180) |- MAP2K1(0.692) <- RAF1(0.998) <- HRAS(0.806) <- SOS2(0.729) <- GRB2(0.940) <- SHC3(0.873) <- MET(0.516)	8	0.0017	84.158786
hsa04810	Regulation of actin cytoskeleton	VCL(1.693) <- PIP5K1A(2.136) <- ROCK2(1.508) <- RHOA(1.025) <- ARHGEF1(1.180) <- GNA12(1.337) <- F2R(1.426)	7	0.0018	76.197615
hsa05218	Melanoma	CDK4(1.030) <- MAPK3(1.278) <- MAP2K2(1.053) <- BRAF(1.146) <- NRAS(1.214) <- IGF1R(1.926) <- FGF20(4.313)	7	0.0018	76.930078
hsa04510	Focal adhesion	BAD(1.180) |- MAP2K1(0.692) <- PAK3(0.732) <- RAC1(0.943) <- VAV2(0.631) <- PIK3CA(0.633) <- HRAS(0.806) <- SOS2(0.729) <- GRB2(0.940) <- SHC3(0.873)	10	0.0018	108.571824
hsa04510	Focal adhesion	BAD(1.180) |- MAP2K1(0.692) <- PAK3(0.732) <- RAC1(0.943) <- VAV2(0.631) <- PIK3CA(0.633) <- HRAS(0.806) <- SOS2(0.729) <- GRB2(0.940) <- SHC3(0.873)	10	0.0018	108.571824
hsa04722	Neurotrophin signaling pathway	ATF4(0.991) <- RPS6KA3(0.945) <- MAPK1(0.731) <- MAP2K1(0.692) <- RAF1(0.998) <- HRAS(0.806) <- SOS2(0.729) <- GRB2(0.940) <- SHC3(0.873)	9	0.0019	90.196122
hsa04722	Neurotrophin signaling pathway	ATF4(0.991) <- RPS6KA3(0.945) <- MAPK1(0.731) <- MAP2K1(0.692) <- RAF1(0.998) <- HRAS(0.806) <- SOS2(0.729) <- GRB2(0.940) <- NTRK2(0.632)	9	0.0019	89.527789
hsa04	Focal	BAD(1.180) |- MAP2K1(0.692) <- PAK3(0.732) <- RAC1(0.943) <-	10	0.0019	108.5

510	adhesion	VAV2(0.631) <- PIK3CA(0.633) <- HRAS(0.806) <- SOS2(0.729) <- GRB2(0.940) <- SHC3(0.873)			71824
hsa04 110	Cell cycle	CCNE2(1.219) <- E2F2(1.978) <- RB1(1.468) |- CCND3(0.984) |- CDKN1A(1.028) <- TP53(1.588) <- CREBBP(2.013)	7	0.0019	71.55 6807
hsa04 810	Regulation of actin cytoskeleton	VCL(1.693) <- PIP5K1A(2.136) <- ROCK2(1.508) |- MAPK1(0.731) <- MAP2K1(0.692) <- RAF1(0.998) <- RRAS(0.863) <- SOS2(0.729) <- FGFR2(0.516)	9	0.002	97.78 9594
hsa04 062	Chemokine signaling pathway	PXN(1.203) <- PTK2(1.059) <- PIK3R1(1.231) <- CSK(1.464) <- GNAI2(1.209) <- CXCR3(1.690) <- GRK4(1.645)	7	0.002	73.17 5252
hsa04 010	MAPK signaling pathway	ATF4(0.991) <- RPS6KA3(0.945) <- MAPK1(0.731) <- MAP2K1(0.692) <- RAF1(0.998) <- RRAS(0.863) <- SOS2(0.729)	7	0.0021	70.09 8281
hsa04 510	Focal adhesion	ITGB3(5.450) <- ACTN2(1.153) <- ZYX(1.157)	3	0.0021	38.86 9796
hsa04 062	Chemokine signaling pathway	RELA(1.165) <- NFKBIB(1.228) <- IKBKB(2.069) <- AKT3(1.232) <- PIK3R1(1.231) <- GNGT1(1.748) <- GNAI2(1.209) <- CXCR3(1.690) <- CXCL5(4.090)	9	0.0021	103.5 76258
hsa04 510	Focal adhesion	BAD(1.180) |- MAP2K1(0.692) <- RAF1(0.998) <- PAK3(0.732) <- RAC1(0.943) <- VAV2(0.631) <- PIK3CA(0.633) <- MET(0.516)	8	0.0022	90.03 5004
hsa04 810	Regulation of actin cytoskeleton	ACTN2(1.153) <- PIP5K1A(2.136) <- ROCK2(1.508) <- RHOA(1.025) <- ARHGEF1(1.180) <- GNA12(1.337) <- F2R(1.426)	7	0.0023	73.75 1855
hsa04 010	MAPK signaling pathway	ATF4(0.991) <- RPS6KA3(0.945) <- MAPK1(0.731) <- MAP2K1(0.692) <- MAP3K1(0.999) <- MAP4K3(0.927)	6	0.0023	61.04 0463
hsa04 110	Cell cycle	CCNE2(1.219) <- E2F2(1.978) <- RB1(1.468) |- CCNA1(0.698) |- CDKN1A(1.028) <- TP53(1.588) <- CREBBP(2.013)	7	0.0023	72.94 9592

hsa05 221	Acute myeloid leukemia	MAPK1(0.731) <- MAP2K1(0.692) <- RAF1(0.998) <- HRAS(0.806) <- SOS2(0.729) <- GRB2(0.940) <- KIT(0.504)	7	0.0023	73.14 0232
hsa04 630	Jak-STAT signaling pathway	PTPN11(0.597) <- JAK1(0.834) <- IL6ST(0.730) |- SOCS3(1.792) <- STAT5A(1.515) <- CREBBP(2.013)	6	0.0024	65.25 621
hsa04 062	Chemokine signaling pathway	MAPK1(0.731) <- MAP2K1(0.692) <- RAF1(0.998) <- HRAS(0.806) <- PIK3CA(0.633) <- GNB5(0.424) <- GNAI1(0.803) <- CCR6(0.793) <- ADRBK1(0.831)	9	0.0024	94.99 1401
hsa04 062	Chemokine signaling pathway	PXN(1.203) <- PTK2(1.059) <- PIK3R1(1.231) <- CSK(1.464) <- GNAI2(1.209) <- CXCR3(1.690) <- ARRB2(1.084)	7	0.0024	72.85 9319
hsa04 110	Cell cycle	CCNE2(1.219) <- E2F2(1.978) <- RB1(1.468) |- CCND3(0.984) |- CDKN1A(1.028) |- CUL1(0.724)	6	0.0024	60.85 9462
hsa04 310	Wnt signaling pathway	CSNK2A2(1.127) <- DVL2(1.888) <- APC2(1.151) <- TBL1X(4.119)	4	0.0025	47.28 9987
hsa04 010	MAPK signaling pathway	ATF4(0.991) <- RPS6KA3(0.945) <- MAPK1(0.731) <- MAP2K1(0.692) <- RAF1(0.998) <- RRAS(0.863) <- RASGRF1(0.486) <- CACNG3(0.562)	8	0.0025	85.19 0914
hsa04 722	Neurotrophin signaling pathway	ATF4(0.991) <- RPS6KA3(0.945) <- MAPK1(0.731) <- MAP2K1(0.692) <- RAF1(0.998) <- HRAS(0.806) <- SOS2(0.729) <- GRB2(0.940) <- NTRK2(0.632)	9	0.0026	89.52 7789
hsa04 910	Insulin signaling pathway	RHOQ(1.970) <- CRKL(1.342)	2	0.0026	24.26 8074
hsa04 062	Chemokine signaling pathway	PXN(1.203) <- PTK2(1.059) <- CSK(1.464) <- GNAI2(1.209) <- CXCR3(1.690) <- CXCL5(4.090)	6	0.0026	69.07 3376
hsa04 810	Regulation of actin	CFL1(1.036) |- LIMK1(0.760) <- PAK3(0.732) <- RAC1(0.943) |- RHOA(1.025) <- ARHGEF1(1.180) <- GNA12(1.337) <- F2R(1.426)	8	0.0027	79.96 1631

	cytoskeleton				
hsa04 730	Long-term depression	ITPR2(1.578) <- PLCB2(1.830) <- GNA11(1.160) <- GRM1(1.808)	4	0.0028	45.41 5559
hsa04 730	Long-term depression	ITPR2(1.578) <- PLCB2(1.830) <- GNA11(1.160) <- GRM1(1.808)	4	0.0028	45.41 5559
hsa04 110	Cell cycle	CCNE2(1.219) <- E2F2(1.978) <- RB1(1.468) |- CCND3(0.984) |- CDKN1A(1.028) <- SMAD3(1.426)	6	0.0028	60.77 1736
hsa04 062	Chemokine signaling pathway	GSK3A(0.891) |- AKT3(1.232) <- PIK3R1(1.231) <- CSK(1.464) <- GNAI2(1.209) <- CXCR3(1.690) <- GRK4(1.645)	7	0.0029	73.64 1918
hsa04 740	Olfactory transduction	PDC(0.421) <- GNAL(0.573) <- OR6A2(0.587) |- ARRB2(1.084)	4	0.003	53.03 801
hsa04 510	Focal adhesion	BAD(1.180) |- RAF1(0.998) <- PAK3(0.732) <- RAC1(0.943) <- VAV2(0.631) <- PIK3CA(0.633) <- MET(0.516)	7	0.003	79.72 1473
hsa04 512	ECM-receptor interaction	SDC3(0.849) <- COL5A2(0.162)	2	0.003	39.34 8289
hsa04 722	Neurotrophin signaling pathway	ATF4(0.991) <- RPS6KA3(0.945) <- MAPK1(0.731) <- MAP2K1(0.692) <- RAF1(0.998) <- HRAS(0.806) <- SOS2(0.729) <- GRB2(0.940)	8	0.0031	79.41 6266
hsa04 010	MAPK signaling pathway	ATF4(0.991) <- RPS6KA3(0.945) <- MAPK1(0.731) |- DUSP7(2.318)	4	0.0031	43.93 0085
hsa04 510	Focal adhesion	ITGB3(5.450) <- PXN(1.203) <- ILK(1.014) <- PIK3R1(1.231) <- PTK2(1.059)	5	0.0031	59.75 4075
hsa04 062	Chemokine signaling pathway	ROCK2(1.508) <- RHOA(1.025) <- VAV1(1.126) <- ITK(1.131) <- PIK3R1(1.231) <- CSK(1.464) <- GNAI2(1.209) <- CXCR3(1.690) <- GRK4(1.645)	9	0.0031	89.48 6796
hsa05	Glioma	PIK3CA(0.633) <- MAP2K1(0.692) <- RAF1(0.998) <- HRAS(0.806) <-	7	0.0032	72.88

214		SOS2(0.729) <- GRB2(0.940) <- SHC3(0.873)			4566
hsa04 510	Focal adhesion	BAD(1.180) |- MAP2K1(0.692) <- RAF1(0.998) <- HRAS(0.806) <- SOS2(0.729) <- GRB2(0.940) <- SHC3(0.873)	7	0.0032	71.09 2223
hsa04 722	Neurotrophin signaling pathway	ATF4(0.991) <- RPS6KA3(0.945) <- MAPK1(0.731) <- MAP2K1(0.692) <- RAF1(0.998) <- HRAS(0.806) <- SOS2(0.729) <- GRB2(0.940)	8	0.0033	79.41 6266
hsa05 214	Glioma	PIK3CA(0.633) <- MAP2K1(0.692) <- RAF1(0.998) <- HRAS(0.806) <- SOS2(0.729) <- GRB2(0.940) <- SHC3(0.873)	7	0.0033	72.88 4566
hsa04 062	Chemokine signaling pathway	GSK3A(0.891) |- AKT3(1.232) <- PIK3R1(1.231) <- CSK(1.464) <- GNAI2(1.209) <- CXCR3(1.690) <- ARRB2(1.084)	7	0.0033	73.32 5985
hsa04 810	Regulation of actin cytoskeleton	ACTN2(1.153) <- PIP5K1A(2.136) <- ROCK2(1.508) |- MAPK1(0.731) <- MAP2K1(0.692) <- RAF1(0.998) <- RRAS(0.863) <- SOS2(0.729) <- FGFR2(0.516)	9	0.0034	95.34 3834
hsa04 810	Regulation of actin cytoskeleton	MYL9(1.851) |- PPP1R12A(0.621) |- ROCK2(1.508) |- MAPK1(0.731) <- MAP2K1(0.692) <- RAF1(0.998) <- RRAS(0.863) <- SOS2(0.729) <- FGFR2(0.516)	9	0.0034	92.63 8567
hsa04 722	Neurotrophin signaling pathway	ATF4(0.991) <- RPS6KA3(0.945) <- MAPK1(0.731) <- MAP2K1(0.692) <- RAF1(0.998) <- HRAS(0.806) <- SOS2(0.729) <- GRB2(0.940)	8	0.0034	79.41 6266
hsa04 510	Focal adhesion	BAD(1.180) |- MAP2K1(0.692) <- RAF1(0.998) <- HRAS(0.806) <- SOS2(0.729) <- GRB2(0.940) <- SHC3(0.873)	7	0.0034	71.09 2223
hsa04 062	Chemokine signaling pathway	ROCK2(1.508) <- RHOA(1.025) <- VAV1(1.126) <- ITK(1.131) <- PIK3R1(1.231) <- CSK(1.464) <- GNAI2(1.209) <- CXCR3(1.690) <- ARRB2(1.084)	9	0.0034	89.17 0863
hsa04 110	Cell cycle	CCNE2(1.219) <- E2F2(1.978) <- RB1(1.468) |- CCNA1(0.698) |- CDKN1A(1.028) <- TP53(1.588) <- CHEK1(1.225) <- ATM(1.296)	8	0.0034	79.09 07
hsa04	Cell cycle	CCNE2(1.219) <- E2F2(1.978) <- RB1(1.468) |- CCNA1(0.698) |-	6	0.0034	62.16

110		CDKN1A(1.028) <- SMAD3(1.426)			4521
hsa04510	Focal adhesion	BAD(1.180) |- MAP2K1(0.692) <- PAK3(0.732) <- RAC1(0.943) <- VAV2(0.631) <- PIK3CA(0.633) <- MET(0.516)	7	0.0035	81.599874
hsa04062	Chemokine signaling pathway	PRKACB(0.903) <- ADCY2(0.724) |- GNAI2(1.209) <- CXCR3(1.690) <- CXCL5(4.090)	5	0.0035	60.103721
hsa04110	Cell cycle	CCNE2(1.219) <- E2F2(1.978) <- RB1(1.468) |- CCND3(0.984) |- CDKN1A(1.028) <- TP53(1.588) <- CHEK1(1.225) <- ATM(1.296)	8	0.0035	77.697914
hsa04722	Neurotrophin signaling pathway	ATF4(0.991) <- RPS6KA3(0.945) <- MAPK1(0.731) <- MAP2K1(0.692) <- RAF1(0.998) <- HRAS(0.806) <- SOS2(0.729) <- GRB2(0.940)	8	0.0036	79.416266
hsa05214	Glioma	PIK3CA(0.633) <- MAP2K1(0.692) <- RAF1(0.998) <- HRAS(0.806) <- SOS2(0.729) <- GRB2(0.940) <- SHC3(0.873)	7	0.0036	72.884566
hsa04510	Focal adhesion	BAD(1.180) |- MAP2K1(0.692) <- RAF1(0.998) <- HRAS(0.806) <- SOS2(0.729) <- GRB2(0.940) <- SHC3(0.873)	7	0.0036	71.092223

Table S5. In GSE12685 (AD) network, the most significant 100-subpathway information. The p-value is the significance of the subpathway based on the statistic S which was introduced in our previous method (PLOS ONE, 2014, 7(4): e31685).

KEGG	Title	Subpathway (linear signaling flow) with fold-change of the disease group over the control group (B <- A: A activates B; B |- A: A represses B)	The number of entries	P-value	S
hsa04740	Olfactory transduction	PDC(0.979) <- PRKX(0.886) <- ADCY3(0.934) <- GNAL(0.760) <- OR10J1(0.868) |- ARRB2(1.044)	6	0	66.995064
hsa04722	Neurotrophin signaling pathway	GSK3B(1.513) |- AKT2(0.856) <- PDK1(0.943) <- PIK3CD(0.576) <- GAB1(0.997) <- SHC2(0.844) <- NTRK1(0.945) <- NTF3(0.784)	8	0	96.491256
hsa04912	GnRH signaling pathway	ELK1(1.035) <- MAPK1(1.401) <- MAP2K1(1.366) <- RAF1(1.101) <- KRAS(2.510) <- SOS1(1.082) <- GRB2(1.273) <- EGFR(1.443) <- HBEGF(1.082)	9	0	103.627666
hsa04060	Cytokine-cytokine receptor interaction	IL22RA1(0.871) <- IL24(0.896)	2	0	27.118582
hsa04062	Chemokine signaling pathway	PRKX(0.886) <- ADCY8(0.869) |- GNAI1(1.165) <- CCR9(1.081) <- ADRBK2(1.501)	5	0	64.541476
hsa04062	Chemokine signaling pathway	GSK3B(1.513) |- AKT2(0.856) <- PIK3CD(0.576) <- LYN(0.893)	4	0	48.199817
hsa04920	Adipocytokine signaling pathway	IRS2(1.321) |- SOCS3(0.803) <- NFKB1(0.875)	3	0	39.188596
hsa04310	Wnt signaling pathway	CSNK2A2(0.975) <- DVL2(0.794) |- CXXC4(1.060)	3	0.0002	34.482663

hsa 04740	Olfactory transduction	PDC(0.979) <- PRKX(0.886) <- ADCY3(0.934) <- GNAL(0.760) <- OR10J1(0.868) |- ADRBK2(1.501)	6	0.0002	70.454532
hsa 04722	Neurotrophin signaling pathway	NFKB1(0.875) <- NFKBIB(0.954) <- AKT2(0.856) <- PDK1(0.943) <- PIK3CD(0.576) <- GAB1(0.997) <- SHC2(0.844) <- NTRK1(0.945) <- NTF3(0.784)	9	0.0002	106.787855
hsa 04722	Neurotrophin signaling pathway	NFKB1(0.875) <- NFKBIB(0.954) <- AKT2(0.856) <- PIK3CD(0.576) <- GAB1(0.997) <- SHC2(0.844) <- NTRK1(0.945) <- NTF3(0.784)	8	0.0002	95.856592
hsa 04080	Neuroactive ligand-receptor interaction	FSHR(0.939) <- FSHB(0.813)	2	0.0002	24.954594
hsa 04062	Chemokine signaling pathway	PRKX(0.886) <- ADCY8(0.869) |- GNAI1(1.165) <- CCR9(1.081) <- XCL2(1.024)	5	0.0002	64.928929
hsa 04062	Chemokine signaling pathway	PRKX(0.886) <- ADCY8(0.869) |- GNAI1(1.165) <- CCR9(1.081) <- ARRB2(1.044)	5	0.0002	61.176319
hsa 04062	Chemokine signaling pathway	GSK3B(1.513) |- AKT2(0.856) <- PIK3CD(0.576) <- GNG4(0.940)	4	0.0002	50.127361
hsa 04110	Cell cycle	CCNE1(1.000) <- E2F1(0.915) <- RB1(0.934) |- CDK4(1.061) |- PCNA(0.967) <- GADD45B(0.761) <- TP53(0.806) <- CHEK1(0.971) <- ATM(0.926)	9	0.0002	94.349724
hsa 05217	Basal cell carcinoma	BMP2(0.907) <- GLI3(0.682)	2	0.0002	26.124382
hsa 049	Adipocytokine signaling pathway	PPARGC1A(1.075) <- STAT3(1.431) <- JAK2(1.016) |- SOCS3(0.803) <- NFKB1(0.875)	5	0.0002	55.8145

20					68
hsa 047 22	Neurotrophin signaling pathway	GSK3B(1.513) |- AKT2(0.856) <- PIK3CD(0.576) <- GAB1(0.997) <- SHC2(0.844) <- NTRK1(0.945) <- NTF3(0.784)	7	0.0003	85.5 439 92
hsa 040 10	MAPK signaling pathway	CDC25B(0.986) <- MAPK12(0.863) <- MAP2K3(0.839) <- MAP3K6(0.776)	4	0.0004	49.2 278 04
hsa 040 10	MAPK signaling pathway	CDC25B(0.986) <- MAPK12(0.863) <- MAP2K3(0.839) |- PPM1A(1.357)	4	0.0004	48.5 251 7
hsa 041 10	Cell cycle	CCNE1(1.000) <- E2F1(0.915) <- RB1(0.934) |- CDK4(1.061) |- PCNA(0.967) <- GADD45B(0.761) <- TP53(0.806) <- CREBBP(0.784)	8	0.0004	86.8 776 44
hsa 049 20	Adipocytokine signaling pathway	NPY(0.929) <- PRKAA2(0.894) |- STAT3(1.431) <- JAK2(1.016) |- SOCS3(0.803) <- NFKB1(0.875)	6	0.0004	65.3 368 37
hsa 040 62	Chemokine signaling pathway	GSK3B(1.513) |- AKT2(0.856) <- PIK3CD(0.576) <- LYN(0.893)	4	0.0005	48.1 998 17
hsa 049 10	Insulin signaling pathway	ACACA(1.354) |- PRKAA2(0.894)	2	0.0006	27.2 709 64
hsa 046 64	Fc epsilon RI signaling pathway	MAPK12(0.863) <- MAP2K3(0.839) <- RAC3(0.892) <- VAV1(0.834) <- SYK(0.919) <- LYN(0.893)	6	0.0006	70.8 112 27
hsa 049 20	Adipocytokine signaling pathway	IRS2(1.321) |- IKBKB(0.936) <- TRAF2(0.835) <- TNFRSF1B(0.960) <- TNF(0.945)	5	0.0006	59.3 175 18

<table>
<tr><td>hsa04010</td><td>MAPK signaling pathway</td><td>ELK4(0.881) <- MAPK12(0.863) <- MAP2K6(0.940) <- MAP3K6(0.776)</td><td>4</td><td>0.0007</td><td>46.047649</td></tr>
<tr><td>hsa04062</td><td>Chemokine signaling pathway</td><td>GSK3B(1.513) |- AKT2(0.856) <- PIK3CD(0.576) <- GNG4(0.940)</td><td>4</td><td>0.0007</td><td>50.127361</td></tr>
<tr><td>hsa04722</td><td>Neurotrophin signaling pathway</td><td>BAD(1.279) |- AKT2(0.856) <- PDK1(0.943) <- PIK3CD(0.576) <- GAB1(0.997) <- SHC2(0.844) <- NTRK1(0.945) <- NTF3(0.784)</td><td>8</td><td>0.0008</td><td>94.624608</td></tr>
<tr><td>hsa04010</td><td>MAPK signaling pathway</td><td>TP53(0.806) <- MAPK12(0.863) <- MAP2K6(0.940) <- MAP3K6(0.776)</td><td>4</td><td>0.0008</td><td>48.382874</td></tr>
<tr><td>hsa04664</td><td>Fc epsilon RI signaling pathway</td><td>MAPK12(0.863) <- MAP2K3(0.839) <- RAC3(0.892) <- VAV1(0.834) <- LCP2(0.921) <- SYK(0.919) <- LYN(0.893)</td><td>7</td><td>0.0008</td><td>80.786959</td></tr>
<tr><td>hsa05200</td><td>Pathways in cancer</td><td>CDK4(1.061) <- MYC(1.039) <- MAPK1(1.401) <- MAP2K1(1.366) <- RAF1(1.101) <- KRAS(2.510) <- SOS1(1.082) <- GRB2(1.273) <- FGFR3(1.283) <- FGF9(1.355)</td><td>10</td><td>0.0009</td><td>117.830291</td></tr>
<tr><td>hsa04722</td><td>Neurotrophin signaling pathway</td><td>BAD(1.279) |- AKT2(0.856) <- PIK3CD(0.576) <- GAB1(0.997) <- SHC2(0.844) <- NTRK1(0.945) <- NTF3(0.784)</td><td>7</td><td>0.0009</td><td>83.677344</td></tr>
<tr><td>hsa04010</td><td>MAPK signaling pathway</td><td>DDIT3(0.842) <- MAPK12(0.863) <- MAP2K3(0.839) <- MAP3K6(0.776)</td><td>4</td><td>0.0009</td><td>51.047135</td></tr>
<tr><td>hsa04010</td><td>MAPK signaling pathway</td><td>DDIT3(0.842) <- MAPK12(0.863) <- MAP2K6(0.940) <- MAP3K6(0.776)</td><td>4</td><td>0.0009</td><td>46.554901</td></tr>
<tr><td>hsa045</td><td>Focal adhesion</td><td>CCND1(0.920) <- JUN(0.976) <- MAPK8(0.921) <- RAC3(0.892) <- VAV1(0.834) <- PIK3CD(0.576) <- ERBB2(0.829) <- PDGFB(0.875)</td><td>8</td><td>0.0009</td><td>94.5160</td></tr>
</table>

10					34
hsa 046 64	Fc epsilon RI signaling pathway	PRKCD(0.821) <- PLCG1(0.918) <- SYK(0.919) <- LYN(0.893)	4	0.0009	44.9 964 05
hsa 052 17	Basal cell carcinoma	GLI3(0.682) <- GLI3(0.682)	2	0.0009	30.8 228 36
hsa 052 17	Basal cell carcinoma	PTCH2(0.938) <- GLI3(0.682)	2	0.0009	26.3 743 65
hsa 043 10	Wnt signaling pathway	CSNK2A2(0.975) <- DVL2(0.794) <- FZD2(0.868) <- WNT7A(0.889) |- WIF1(1.208)	5	0.001	60.2 975 17
hsa 047 40	Olfactory transduction	CLCA1(0.790) <- CNGB1(0.886) <- ADCY3(0.934) <- GNAL(0.760) <- OR10J1(0.868) |- ARRB2(1.044)	6	0.001	71.2 367
hsa 046 30	Jak-STAT signaling pathway	PIM1(0.935) <- STAT2(0.964) <- JAK3(0.861) <- IL2RA(0.760) |- CBLB(1.202)	5	0.001	62.4 522 62
hsa 041 10	Cell cycle	CCNE1(1.000) <- E2F1(0.915) <- RB1(0.934) |- CDK4(1.061) |- PCNA(0.967) <- GADD45B(0.761) <- TP53(0.806) <- ATM(0.926)	8	0.001	84.8 028 42
hsa 043 10	Wnt signaling pathway	NFATC3(0.904) <- PPP3CC(0.622) <- PLCB3(0.891) <- FZD2(0.868) <- WNT5B(0.776)	5	0.0011	65.1 340 28
hsa 040 10	MAPK signaling pathway	TP53(0.806) <- MAPK12(0.863) <- MAP2K3(0.839) <- MAP3K6(0.776)	4	0.0011	52.8 751 08

hsa 040 10	MAPK signaling pathway	TP53(0.806) <- MAPK12(0.863) <- MAP2K3(0.839) |- PPM1A(1.357)	4	0.0011	52.1 724 74
hsa 040 62	Chemokine signaling pathway	GSK3B(1.513) |- AKT2(0.856) <- PIK3CD(0.576) <- GNG4(0.940)	4	0.0011	50.1 273 61
hsa 040 62	Chemokine signaling pathway	GSK3B(1.513) |- AKT2(0.856) <- PIK3CD(0.576) <- LYN(0.893)	4	0.0011	48.1 998 17
hsa 046 62	B cell receptor signaling pathway	LILRB3(0.717) <- PTPN6(0.880)	2	0.0011	25.8 012 36
hsa 043 60	Axon guidance	CFL1(1.157) |- LIMK1(0.896) <- PAK4(0.871) <- RAC3(0.892) <- PLXNA3(0.954) <- FES(0.841)	6	0.0011	68.6 817 93
hsa 049 20	Adipocytokine signaling pathway	AGRP(0.999) <- PRKAA2(0.894) |- STAT3(1.431) <- JAK2(1.016) |- SOCS3(0.803) <- NFKB1(0.875)	6	0.0011	64.4 406 51
hsa 047 40	Olfactory transduction	CLCA1(0.790) <- CNGB1(0.886) <- ADCY3(0.934) <- GNAL(0.760) <- OR10J1(0.868) |- ADRBK2(1.501)	6	0.0012	74.6 961 68
hsa 045 12	ECM-receptor interaction	GP6(0.978) <- COL3A1(0.865)	2	0.0012	22.6 301 95
hsa 041 10	Cell cycle	CCNE1(1.000) <- E2F1(0.915) <- RB1(0.934) |- CDK4(1.061) |- PCNA(0.967) <- GADD45B(0.761) <- TP53(0.806) <- PRKDC(0.931)	8	0.0012	84.7 124 98
hsa 047	Neurotrophin signaling pathway	SORT1(0.977) <- NGFR(0.965) <- NTF3(0.784)	3	0.0013	36.1 452

22					29
hsa 046 30	Jak-STAT signaling pathway	CCND1(0.920) <- STAT2(0.964) <- JAK3(0.861) <- IL2RA(0.760) |- CBLB(1.202)	5	0.0013	60.8 208 79
hsa 041 30	SNARE interactions in vesicular transport	STX6(0.842) <- VAMP4(0.938)	2	0.0014	23.3 702 53
hsa 052 00	Pathways in cancer	LEF1(0.920) <- JUP(0.889) <- RARA(0.780)	3	0.0014	35.5 922 64
hsa 052 00	Pathways in cancer	LEF1(0.920) <- JUP(0.889) <- RARA(0.780)	3	0.0014	35.5 922 64
hsa 047 22	Neurotrophin signaling pathway	RIPK2(0.881) <- NGFR(0.965) <- NTF3(0.784)	3	0.0014	37.1 608 4
hsa 040 10	MAPK signaling pathway	DDIT3(0.842) <- MAPK12(0.863) <- MAP2K3(0.839) |- PPM1A(1.357)	4	0.0014	50.3 445 01
hsa 040 10	MAPK signaling pathway	TP53(0.806) <- MAPK12(0.863) |- PTPRR(1.198)	3	0.0014	38.3 591 32
hsa 040 12	ErbB signaling pathway	MYC(1.039) <- MAPK1(1.401) <- MAP2K1(1.366) <- RAF1(1.101) <- KRAS(2.510) <- SOS1(1.082) <- GRB2(1.273) <- EGFR(1.443) <- HBEGF(1.082)	9	0.0014	102. 987 837
hsa 040 62	Chemokine signaling pathway	CRKL(1.487) <- PTK2B(1.421) <- FGR(1.063) <- GNAI1(1.165) <- CCR9(1.081) <- XCL2(1.024)	6	0.0014	73.3 299 19

hsa 052 21	Acute myeloid leukemia	LEF1(0.920) <- JUP(0.889) <- RARA(0.780)	3	0.0014	35.5 922 64
hsa 052 21	Acute myeloid leukemia	LEF1(0.920) <- JUP(0.889) <- RARA(0.780)	3	0.0014	35.5 922 64
hsa 052 00	Pathways in cancer	MMP9(1.139) <- MYC(1.039) <- MAPK1(1.401) <- MAP2K1(1.366) <- RAF1(1.101) <- KRAS(2.510) <- SOS1(1.082) <- GRB2(1.273) <- FGFR3(1.283) <- FGF9(1.355)	10	0.0015	114. 929 854
hsa 043 40	Hedgehog signaling pathway	PTCH2(0.938) <- GLI3(0.682) |- GSK3B(1.513)	3	0.0015	39.1 767 93
hsa 046 64	Fc epsilon RI signaling pathway	PRKCD(0.821) <- PLCG1(0.918) <- LCP2(0.921) <- SYK(0.919) <- LYN(0.893)	5	0.0015	55.1 333 21
hsa 043 10	Wnt signaling pathway	CAMK2B(3.657) <- PLCB1(2.650) <- FZD7(1.078)	3	0.0016	51.3 161 02
hsa 046 60	T cell receptor signaling pathway	NFATC3(0.904) <- MAPK12(0.863) <- ZAP70(0.862) |- CBLB(1.202)	4	0.0016	50.7 215 82
hsa 049 16	Melanogenesis	MITF(0.933) <- CREB1(0.863) <- PRKX(0.886)	3	0.0016	32.8 667 21
hsa 040 10	MAPK signaling pathway	DDIT3(0.842) <- MAPK12(0.863) |- PTPRR(1.198)	3	0.0016	36.5 311 59
hsa 046	Jak-STAT signaling pathway	PTPN11(0.861) <- JAK3(0.861) <- IL2RA(0.760) |- CBLB(1.202)	4	0.0016	50.9 422

30					86
hsa 040 60	Cytokine-cytokine receptor interaction	LTBR(0.887) <- LTA(0.894)	2	0.0016	24.5 077 42
hsa 049 20	Adipocytokine signaling pathway	IRS2(1.321) |- IKBKB(0.936) <- PRKCQ(0.934)	3	0.0016	34.4 985 55
hsa 049 30	Type II diabetes mellitus	INS(0.796) <- CACNA1G(0.859) <- ABCC8(0.865)	3	0.0017	37.3 570 94
hsa 046 30	Jak-STAT signaling pathway	SPRED2(1.638) <- SOS1(1.082)	2	0.0017	25.1 215 25
hsa 049 12	GnRH signaling pathway	ELK1(1.035) <- MAPK1(1.401) <- MAP2K1(1.366) <- RAF1(1.101) <- KRAS(2.510) <- SOS1(1.082) <- GRB2(1.273) <- EGFR(1.443)	8	0.0017	93.5 319 22
hsa 045 12	ECM-receptor interaction	SDC1(0.865) <- COL3A1(0.865)	2	0.0017	27.3 756 53
hsa 040 10	MAPK signaling pathway	ELK4(0.881) <- MAPK12(0.863) <- MAP2K3(0.839) <- MAP3K6(0.776)	4	0.0018	50.5 398 83
hsa 040 10	MAPK signaling pathway	NFKB2(0.871) <- MAP3K14(0.793) <- MAP3K1(0.896) <- RAC3(0.892)	4	0.0018	48.1 831 6
hsa 045 10	Focal adhesion	ITGB3(0.678) <- PARVB(0.928)	2	0.0018	25.1 872 23

hsa 043 10	Wnt signaling pathway	PRKCG(0.915) <- PLCB3(0.891) <- FZD2(0.868) <- WNT5B(0.776)	4	0.0019	46.9 625 87
hsa 052 00	Pathways in cancer	PGF(1.112) <- MYC(1.039) <- MAPK1(1.401) <- MAP2K1(1.366) <- RAF1(1.101) <- KRAS(2.510) <- SOS1(1.082) <- GRB2(1.273) <- FGFR3(1.283) <- FGF9(1.355)	10	0.0019	114. 851 537
hsa 040 10	MAPK signaling pathway	TP53(0.806) <- MAPK12(0.863) <- MAP2K6(0.940) |- PPM1A(1.357)	4	0.0019	47.5 519 85
hsa 049 10	Insulin signaling pathway	ACACA(1.354) <- SREBF1(1.316) <- PRKCI(1.889) <- PIK3R1(1.809) <- IRS2(1.321) |- IKBKB(0.936)	6	0.0019	82.9 788 93
hsa 049 10	Insulin signaling pathway	PDE3B(0.854) <- PRKX(0.886)	2	0.0019	25.1 785 35
hsa 045 10	Focal adhesion	ITGB3(0.678) <- COL3A1(0.865)	2	0.0019	26.4 571 12
hsa 052 00	Pathways in cancer	CDK4(1.061) <- MYC(1.039) <- MAPK1(1.401) <- MAP2K1(1.366) <- RAF1(1.101) <- KRAS(2.510) <- SOS1(1.082) <- GRB2(1.273) <- IGF1R(1.360)	9	0.002	106. 050 489
hsa 046 30	Jak-STAT signaling pathway	PTPN11(0.861) <- JAK3(0.861) <- IL2RA(0.760) |- SOCS5(1.255) <- STAT4(1.109) |- PIAS2(0.927)	6	0.002	70.3 801 94
hsa 040 12	ErbB signaling pathway	ELK1(1.035) <- MAPK1(1.401) <- MAP2K1(1.366) <- RAF1(1.101) <- KRAS(2.510) <- SOS1(1.082) <- GRB2(1.273) <- EGFR(1.443) <- HBEGF(1.082)	9	0.002	103. 627 666
hsa 046	NOD-like receptor signaling pathway	PSTPIP1(0.788) <- MEFV(0.930)	2	0.0021	21.9 982

21					97
hsa 040 10	MAPK signaling pathway	NFKB2(0.871) <- IKBKB(0.936) <- MAP3K1(0.896) <- RAC3(0.892)	4	0.0021	47.9 645 01
hsa 040 10	MAPK signaling pathway	TP53(0.806) <- MAPK12(0.863) |- DUSP6(1.315)	3	0.0021	38.2 668 5
hsa 046 30	Jak-STAT signaling pathway	AKT3(1.297) <- PIK3R1(1.809) <- JAK1(1.208) |- SOCS3(0.803) <- STAT2(0.964) <- CREBBP(0.784)	6	0.0021	74.5 818 9
hsa 041 44	Endocytosis	LDLR(0.716) <- LDLRAP1(0.866)	2	0.0021	26.9 848 51
hsa 052 00	Pathways in cancer	CDK4(1.061) <- MYC(1.039) <- MAPK1(1.401) <- MAP2K1(1.366) <- RAF1(1.101) <- KRAS(2.510) <- SOS1(1.082) <- GRB2(1.273) <- KIT(1.118) <- KITLG(1.073)	10	0.0022	111. 803 333
hsa 046 30	Jak-STAT signaling pathway	AKT2(0.856) <- PIK3CD(0.576) <- JAK3(0.861) <- IL2RA(0.760) |- SOCS5(1.255) <- STAT4(1.109) |- CISH(0.867)	7	0.0022	87.0 886 71

Supplementary Figure legends

Figure S1. The network generated from GSE4107 (CRC) dataset by our previous systems biology approach. The more red in node color, the higher expression in the CRC patients over controls. The more green in node color, vice versa.

Figure S2. The network generated from GSE12685 (AD) dataset by our previous systems biology approach. The more red in node color, the higher expression in the AD patients over controls. The more green in node color, vice versa.

Figure S3. The network generated from GSE1297 (AD) dataset by our previous systems biology approach. The more red in node color, the higher expression in the AD patients over controls. The more green in node color, vice versa.

Figure S1.

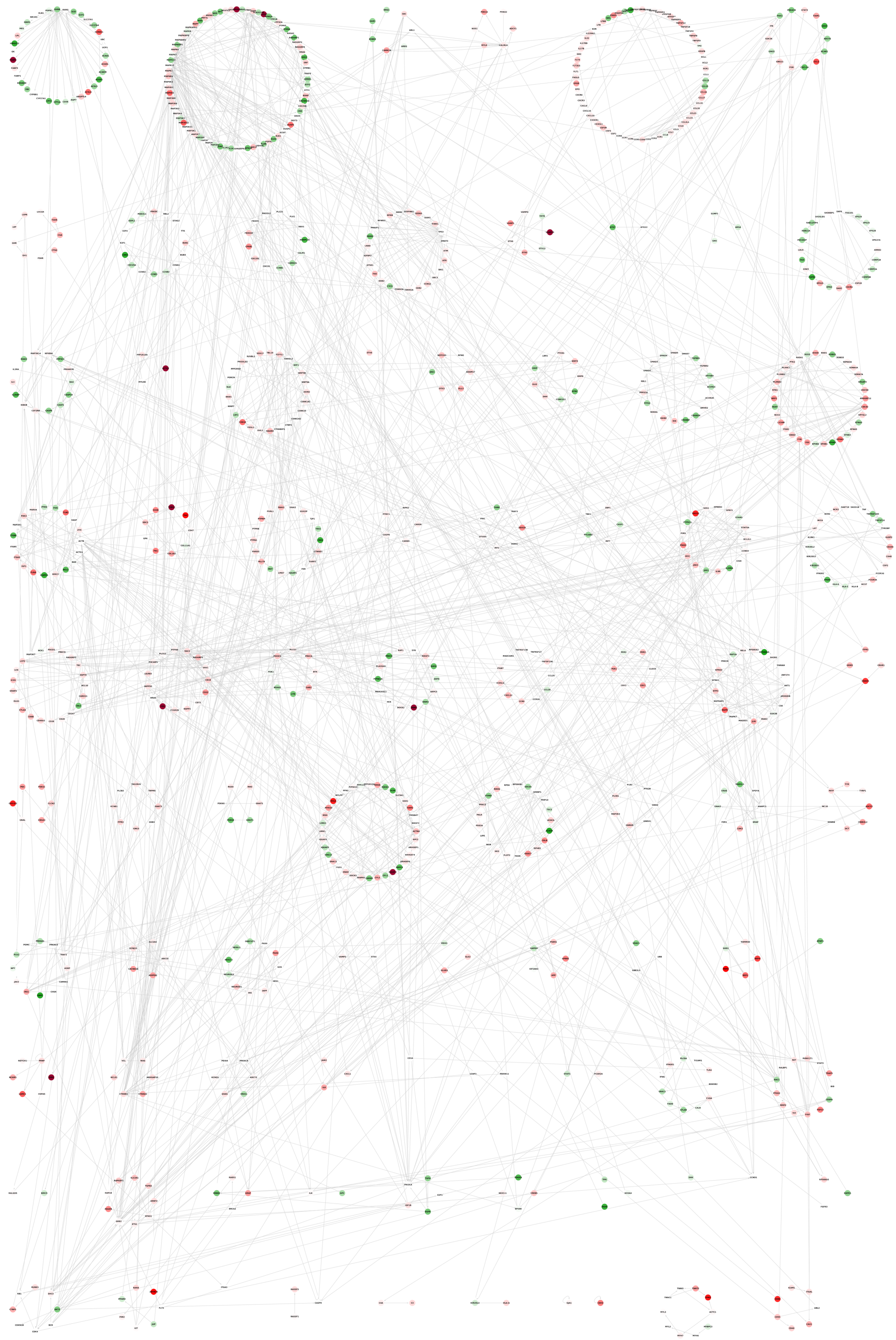

Figure S2.

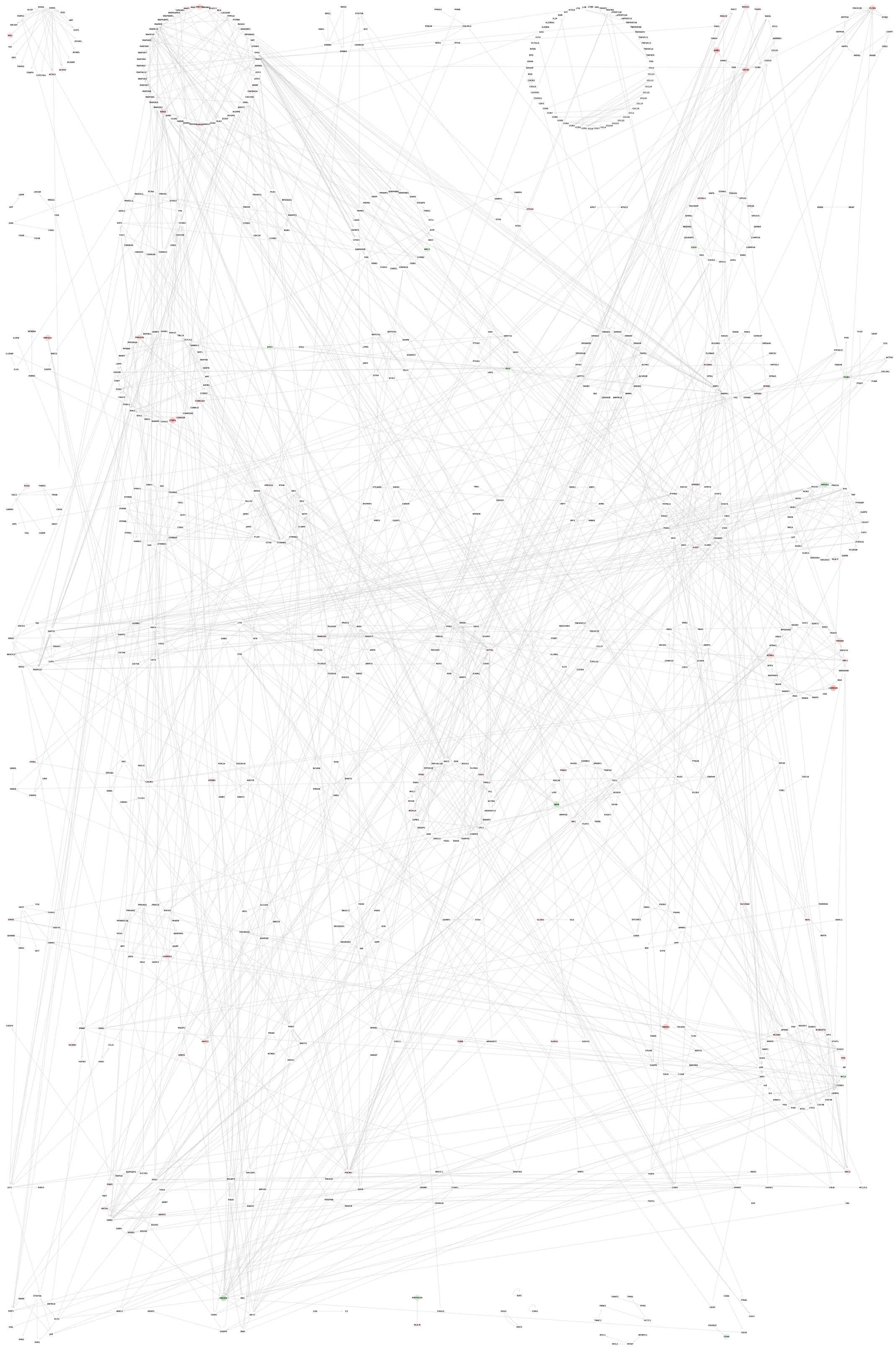

Figure S3.

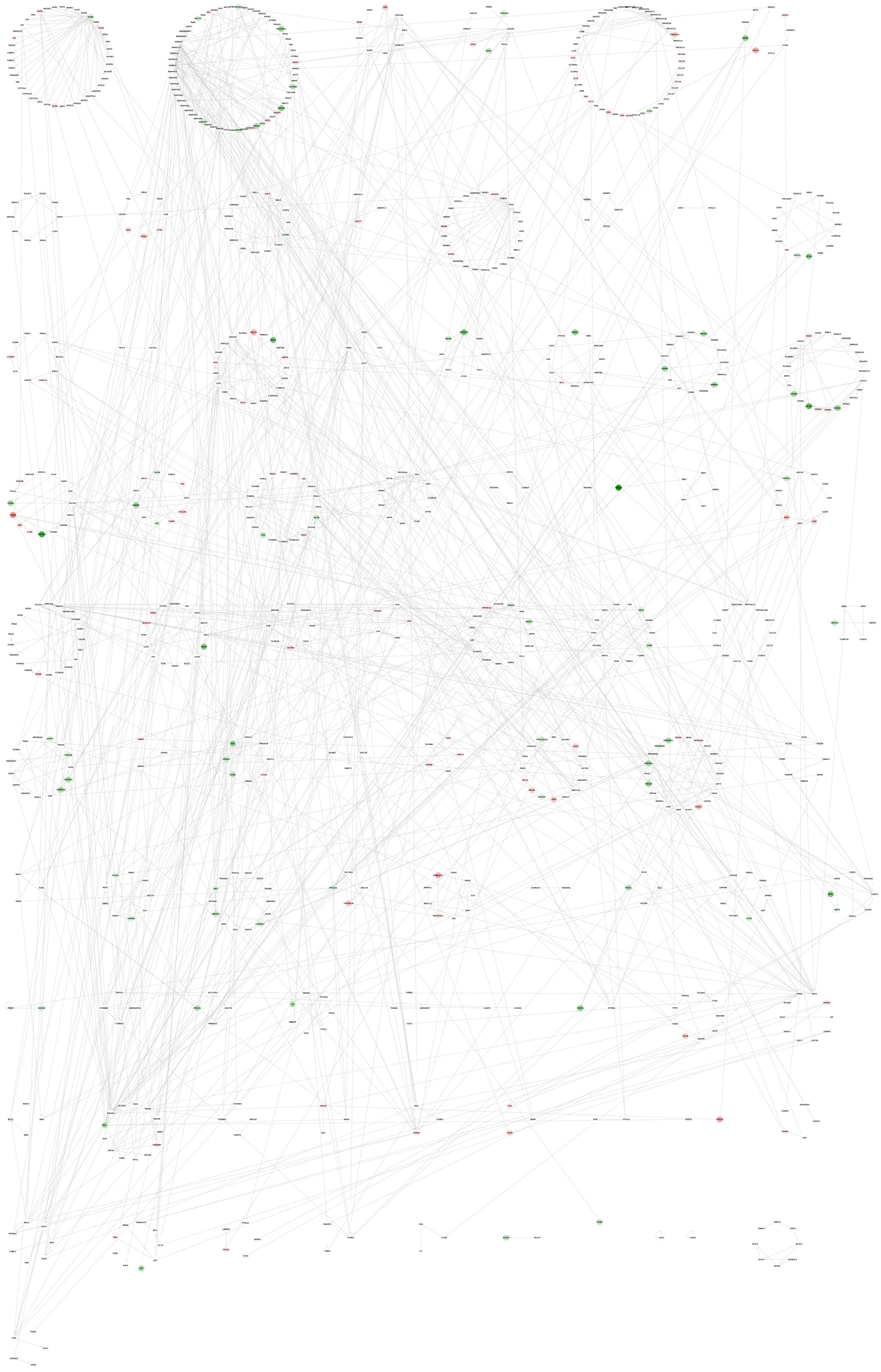

References

1. Hong Y, Ho KS, Eu KW, Cheah PY: **A susceptibility gene set for early onset colorectal cancer that integrates diverse signaling pathways: implication for tumorigenesis.** *Clin Cancer Res* 2007, **13:**1107-1114.
2. Williams C, Mehrian Shai R, Wu Y, Hsu YH, Sitzer T, Spann B, McCleary C, Mo Y, Miller CA: **Transcriptome analysis of synaptoneurosomes identifies neuroplasticity genes overexpressed in incipient Alzheimer's disease.** *PLoS One* 2009, **4:**e4936.
3. Blalock EM, Geddes JW, Chen KC, Porter NM, Markesbery WR, Landfield PW: **Incipient Alzheimer's disease: microarray correlation analyses reveal major transcriptional and tumor suppressor responses.** *Proc Natl Acad Sci U S A* 2004, **101:**2173-2178.
